# Imaging mass cytometry reveals functional and immunological changes during type 1 diabetes progression in human pancreata

**DOI:** 10.1038/s42255-026-01559-z

**Published:** 2026-07-02

**Authors:** Nathan Steenbuck, Nicolas Damond, Stefanie Engler, Irina Kusmartseva, Amanda L. Posgai, Denise M. Drotar, MacKenzie D. Williams, Natalie de Souza, Todd M. Brusko, Maigan A. Brusko, Clive H. Wasserfall, Mark A. Atkinson, Bernd Bodenmiller

**Affiliations:** 1 Department of Quantitative Biomedicine, University of Zurich, Zurich, Switzerland; 2 Life Science Zurich Graduate School, ETH Zurich and University of Zurich, Zurich, Switzerland; 3 Institute of Molecular Health Sciences, ETH Zurich, Zurich, Switzerland; 4 Department of Pathology, Immunology and Laboratory Medicine, College of Medicine, University of Florida Diabetes Institute, Gainesville, FL USA; 5 Institute for Molecular Systems Biology, ETH Zurich, Zurich, Switzerland; 6 Department of Pediatrics, College of Medicine, University of Florida Diabetes Institute, Gainesville, FL USA

**Keywords:** Autoimmunity, Proteomic analysis, Type 1 diabetes, Metabolism

## Abstract

The pathogenesis of type 1 diabetes, particularly at autoantibody-positive preclinical stages, remains poorly understood, in part due to limited sample availability. Here we show imaging mass cytometry data of pancreas samples from 88 organ donors, including 28 single and 10 multiple autoantibody-positive donors. We imaged 16 million single-cells using 79 antibodies to characterize β-cell states and the islet–immune interface, correcting for relevant covariates. We identified interactions between pro-inflammatory macrophages and exhausted-like (PD1^+^TIM3^+^) T cells. These interactions were characteristic of early disease and of insulitic islets, indicating a key role of macrophages in disease development. β-cells showed loss of IAPP in preclinical disease, insulitic interferon signatures and no increase in three measured endoplasmic reticulum stress markers in disease samples relative to control. Multiple immune cell subtypes were associated with young age and insulitis, potentially contributing to greater disease severity in younger patients. Our data present a resource describing early type 1 diabetes progression and reveal potentially clinically actionable features before extensive β-cells loss.

## Abstract (web summary)

This Resource presents imaging mass cytometry data of islets, surrounding exocrine tissue and infiltrating immune cells from donors with type 1 diabetes across disease stages, highlighting functional and immunological changes during disease progression in humans.

## Main

Type 1 diabetes (T1D) is a chronic autoimmune disease characterized by the selective loss of insulin-producing pancreatic β-cells^1^. Despite the high global disease burden, neither a prevention nor a cure has been identified in part due to incomplete understanding of T1D pathogenesis^2,3^. β-cell loss is triggered by an unknown autoimmune event that results in autoantibodies that target β-cell antigens. These can emerge years before clinical symptoms and offer valuable prognostic insights. The presence of a single autoantibody (sAAb+) indicates a lifetime T1D risk of approximately 15%, whereas two or more autoantibodies (mAAb+) signal a risk of around 85% (ref. ^4^). mAAb+ subjects are classified as stage 1, mAAb+ and dysglycemic subjects as stage 2^5^ and subjects with substantial loss of β-cell mass as having stage 3 disease. Symptoms at diagnosis and time to complete β-cell loss are highly variable between patients^5,6^. Understanding the biological processes driving T1D progression, particularly during the sAAb+ and mAAb+ stages, will be essential to guide development of therapeutic strategies that preserve endogenous β-cells.

T1D can be considered mainly a disease of the adaptive immune system^7^, given the importance of autoantibodies, β-cell destruction by autoreactive T cells^8,9^ and the partial success of T cell-targeting therapeutics in delaying T1D onset^10–12^. Mouse studies indicate that components of the innate immune system, particularly dendritic cells (DCs) and macrophages, influence initiation^13^ and progression of the disease^14,15^, but the role of the innate immune system in the human pancreas remains poorly defined^16,17^. T1D is also associated with dysregulation in the endocrine compartment, including MHC-I hyperexpression^18^, secretion of and response to pro-inflammatory cytokines^19^ and endoplasmic reticulum (ER) stress in β-cells^20–22^. Given the wide range of disease states at the pancreatic islet–immune interface and heterogeneity associated with age, clinical presentation and intervention response^23^, a comprehensive characterization of human pancreata by single-cell, spatially resolved and multiplexed modalities at large scale and across ages is needed.

So far, only a few spatial multiplexed protein imaging studies have been conducted on human T1D samples. These were limited by small cohort sizes, with just two autoantibody positive donors, in particular, evaluated so far^6,24–26^. To study the earliest steps of T1D development, we used imaging mass cytometry (IMC) on pancreatic sections from 88 organ donors spanning the entire spectrum of T1D progression, including 28 sAAb+ and 10 mAAb+ donors, and controls without T1D. We used two 45-plex antibody panels to extensively characterize the islet–immune interface. Using these IMC data, we identified IAPP loss, pro-inflammatory myeloid phenotypes and PD1^+^ T cells as critical indicators of progression in mAAb+ donors, suggesting that these are potential therapeutic targets. Further, we integrated our data with clinical co-variates to describe disease phenotypes associated with age.

## Results

### Highly multiplex imaging of human T1D samples

**Fig. 1:**
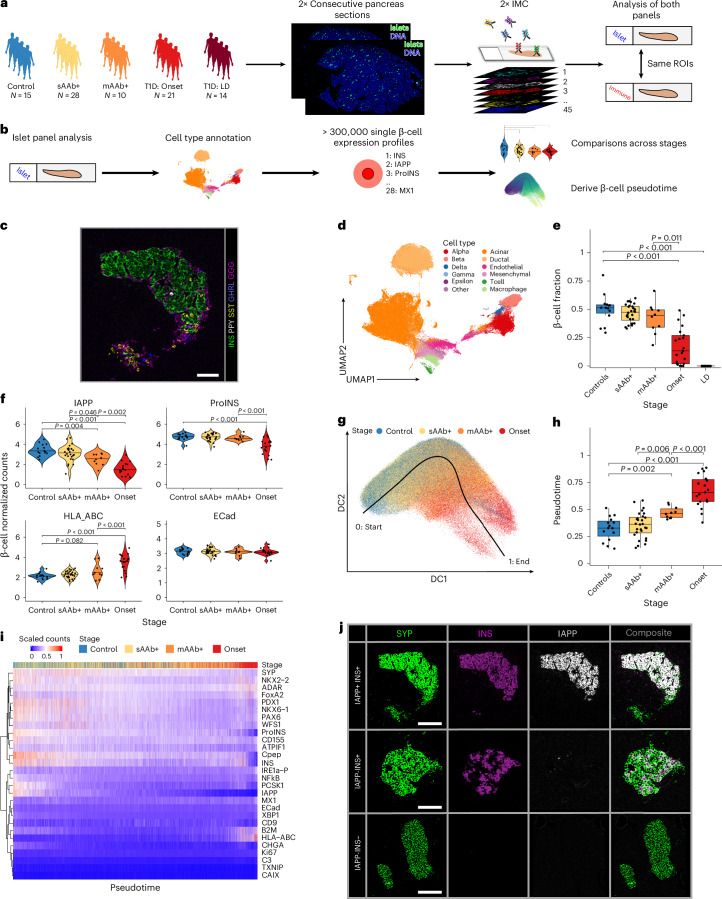
IAPP is downregulated and MHC-I is upregulated along the β-cell pseudotime. **a**, A schematic showing the experimental design of our study. Pancreatic samples from 88 cadaveric organ donors were analysed. **b**, Data analysis using the islet antibody panel. **c**, Representative pancreatic islet image from a non-diabetic control donor. INS, GCG, SST, PPY and GHRL mark β-, α-, δ-, γ-, ε-cells, respectively. Scale bar, 75 µm. **d**, Uniform manifold approximation and projection of a subset of 190,000 cells coloured by cell type. **e**, Fraction of β-cells per islet across disease stage groups (each dot shows mean per sample). **f**, Expression of indicated β-cell markers across disease stages (each dot shows mean per donor across ROIs). The centre line indicates the median. **g**, Trajectory inferred using slingshot from expression profiles of single β-cells projected onto a diffusion map; coloured by donor stage. **h**, Average β-cell pseudotime across disease stages (each dot shows mean per donor). **i**, A heat map of mean β-cell marker expression values across ROIs (min–max scaled, shown in columns). ROIs are ordered by pseudotime averages. **j**, Representative images of samples from control and disease stages stained for SYP (islets), IAPP and INS. Scale bar, 100 µm. Tests: non-significant tests are not shown. Differential expression was calculated using linear mixed-effects models (two-sided Wald *t*-test with Satterthwaite approximation) and differential abundances using edgeR (two-sided empirical Bayes quasi-likelihood *F*-tests). For boxplots in **e** and **h**, the centre line indicates the median, box bounds the interquartile range (IQR) and whiskers 1.5× IQR. *P* values are FDR adjusted. For **e**, **f** and **h**: controls: *N* = 15; sAAb+: *N* = 28; mAAb+: *N* = 10; Onset: *N* = 21 for **e** or *N* = 18 for **f** and **h**; LD: *N* = 15 for **e**.

**Extended Data Fig. 1:**
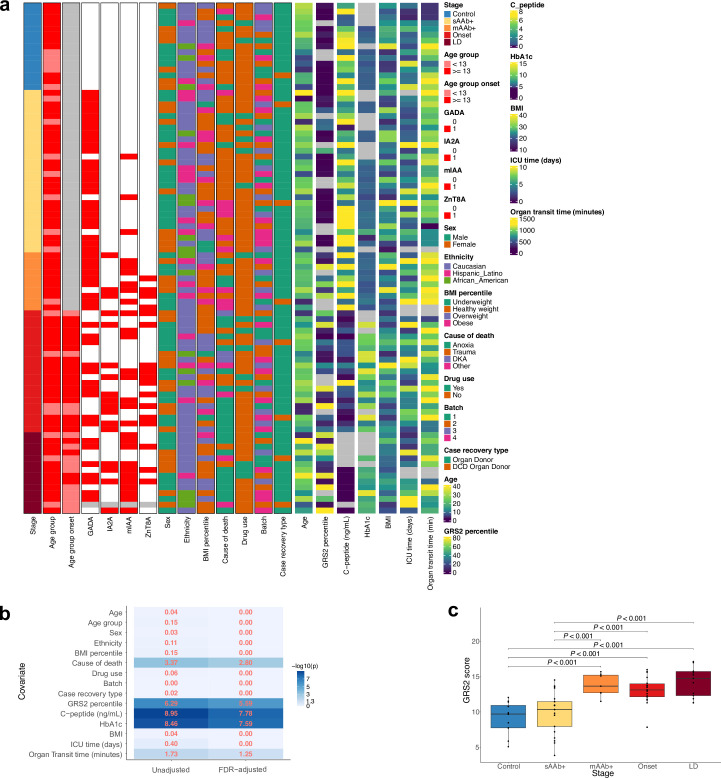
IMC cohort is matched by BMI, age and sex. **a,** Heatmap of clinical and technical covariates of the organ donors in our cohort. Values are also shown in Supplementary Table 1. Gray elements indicate not applicable values. **b,** Association of covariates to disease stages. FDR adjusted significances were calculated using Kruskal-Wallis tests for numerical variables and χ^2^ tests for categorical variables (both two-sided). **c,** T1D Genetic risk scores (GRS2) of donors across stages (each dot shows one donor). Center line defines the median, box bounds the IQR, and whiskers extend to 1.5x the IQR. Data are as shown in (**a**) and reflect population-level trends of T1D risk. Tests: two-sided Welch *t*-test with Bonferroni correction. Non-significant tests are not shown.

Studies of early T1D progression have been limited by sample availability^3^. For this study, we obtained pancreatic tissue sections from 88 cadaveric organ donors across the entire spectrum of T1D risk and progression from the Network for Pancreatic Organ donors with Diabetes (nPOD) biorepository (Fig. 1a and Supplementary Table 1). Included were samples from sAAb+ (*N* = 28) and mAAb+ (*N* = 10) donors, recent-onset (≤2 years) (Onset) T1D donors (*N* = 21) and long-duration (≥3 years) (LD) T1D donors (*N* = 14), as well as controls (*N* = 15), with disease stages matched in terms of multiple covariates, including age, sex and BMI (Extended Data Fig. 1a). Cause of death and organ transit time were the only measured covariates that were not balanced across disease stages (Extended Data Fig. 1b), and we ensured that these did not affect our results (see below). Other differences between disease stages (higher genetic risk and insulin treatment in diabetic donors) reflect population-level and clinical reality (Extended Data Fig. 1a,c) and the challenges inherent to studies of human samples (Discussion). Enabling the study of potential age-associated endotypes^23^, 11 donors were less than 7 years of age, and 9 were between 7 and 12 years of age. For a more robust statistical analysis, we pooled donors into groups of <13 years of age (*N* = 20) and ≥13 years of age (*N* = 68).

**Extended Data Fig. 2:**
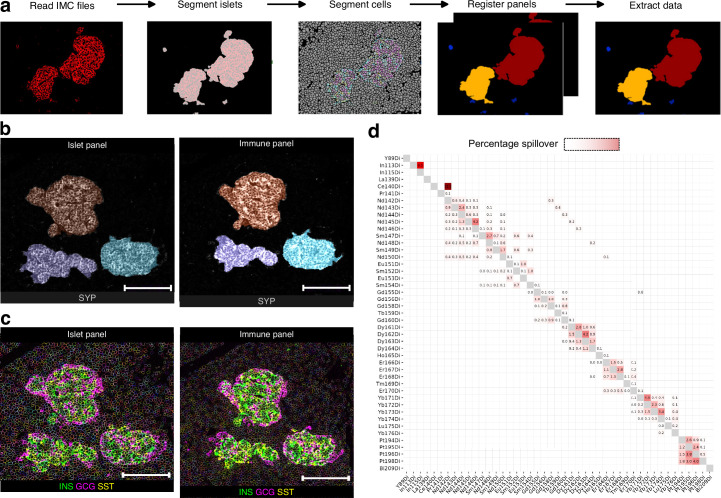
IMC pre-processing workflow. **a,** Schematic of the implemented pre-processing pipeline. **b,** Islet masks of acquired images from serial sections stained with the islet and immune panel. Scale bar is 100 µm. **c,** Cell masks of acquired images from serial sections stained with the islet and immune panel. Scale bar is 100 µm. **d,** Representative spillover matrix.

To extensively analyse the endocrine and autoimmune compartments as well as the islet–immune interface, we designed two IMC antibody panels targeting a total of 79 unique protein markers (Supplementary Table 2 and 3). Specificity and association to T1D progression were tested in a pilot IMC study (*N* = 130 antibodies) applied to control (*N* = 4), sAAb+ (*N* = 4), mAAb+ (*N* = 4) and Onset T1D donors (*N* = 4) (Supplementary Table 4–8). We provide this 130-plex dataset. Using these two optimized panels, we imaged two 4-µm-thick consecutive sections from each donor. Because the speed of IMC data acquisition limits whole-slide imaging, we selected regions of interest (ROIs) based on immunofluorescence imaging of islet (CD99) and immune markers (CD45RO/CD45RA; CD3e). We chose ROIs to comprise islets with proximal exocrine tissue, with the goal of capturing both the endocrine state and the immune compartment. We then stained the sections with metal-labelled primary antibodies and acquired 75 selected ROIs per section by IMC. The same locations were imaged in consecutive sections using the second antibody panel. We segmented cells and islets in each image (Extended Data Fig. 2), with islets defined as SYP^high^ objects of more than 50 µm^2^ (~8 µm diameter) (Methods). Our ROI and islet segmentation strategy results in an under-sampling of small islets, but this effect was small (Supplementary Fig. 1a–d; Discussion). After stringent quality control and signal spillover correction (Extended Data Fig. 2d), our dataset comprised 7,025 ROIs for each antibody panel, 10,413 islets and >16 million cells. To our knowledge, this represents, by far, the largest multiplexed imaging effort of T1D samples so far. Full data are available via Zenodo^27^ and annotated image data (for example, ‘insulitic’) is available in an easily browsable format at Pancreatlas (https://www.pancreatlas.org/)^28^.

### β-cells and functional β-cell markers are lost during disease progression

**Extended Data Fig. 3:**
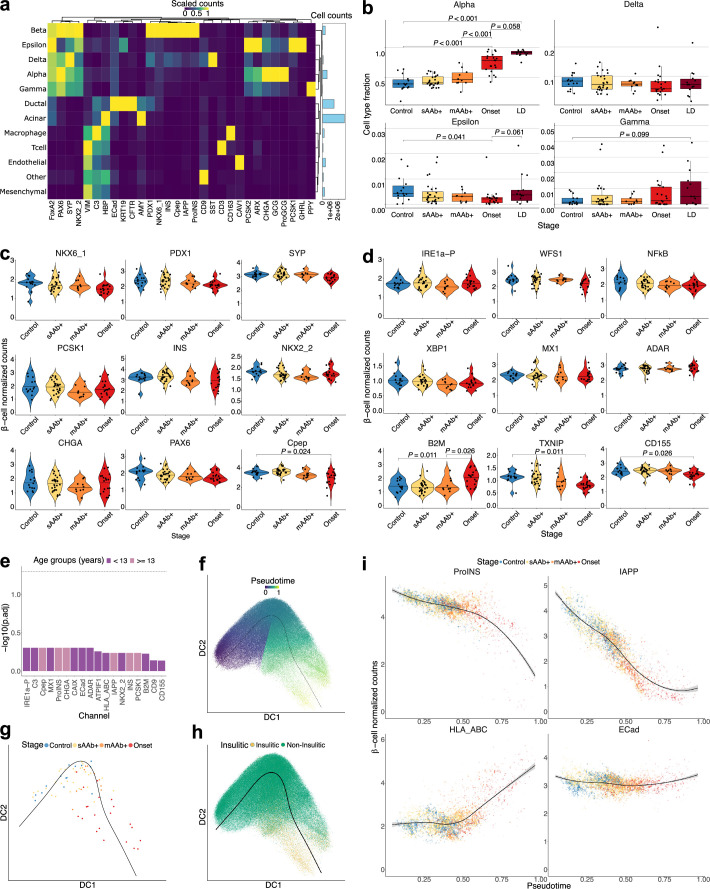
β-cell expression profiles change with disease stage. **a,** Heatmap of indicated marker expression in annotated cell types; barplots indicate total counts. **b**, Fractions of α-, δ-, ε-, and γ-cells per islet across disease stages (each dot, mean per donor across ROIs). **c, d,** Asinh-normalized counts across disease stages of **(c)** β-cell lineage/ islet markers and **(d)** functional markers (each dot, mean per donor across ROIs). The center line indicates the median, box bounds the IQR, and whiskers extend to 1.5x the IQR. **e,** β-cell marker expression compared between samples from Onset donors <13 years (N = 4) and ≥13 years (N = 17). Colouring indicates the age group with higher expression levels. Only channels with an unadjusted *P*-value < 0.5 are shown; no tests were significant. **f-h,** Diffusion map-embedded β-cells coloured by (**f**) slingshot pseudotime, (**g**) donor-averaged pseudotime, and (**h**) insulitic status. **i**, β-cell marker expression (ROI means) along pseudotime with LOWESS fits and 95% CIs. Tests: Non-significant tests are not shown. Differential abundance was tested using edgeR (two-sided empirical Bayes quasi-likelihood *F*-test) and differential expression by linear mixed-effects models (two-sided Wald *t*-tests with Satterthwaite approximation). **(b, c, d)**: controls: N = 15; sAAb+: N = 28; mAAb+: N = 10; Onset: **(b)** N = 21 or **(c, d)** N = 18; LD: (**b**) N = 14.

Our islet antibody panel was designed to study dysregulation in the endocrine compartment (Fig. 1b) and included antibodies to lineage markers of islet cells, as well as to functional markers of ER stress (WFS-1, IRE1α-P and XBP1), the interferon (IFN) response (MX1 and ADAR), islet inflammation (B2M and HLA-ABC) and redox and inflammatory signalling (TXNIP, NF-κB and CD155) (Supplementary Table 3). We annotated cells by clustering single-cell lineage marker expression profiles, annotating the resulting clusters as abundant (α, β and δ) and rare (ε and γ) endocrine cell types (Fig. 1c) and subsequently labelling all non-endocrine cells as acinar, ductal, endothelial, mesenchymal, macrophage, T cell or ‘other’ (Fig. 1d and Extended Data Fig. 3a). The last category probably encompasses immune cells such as neutrophils. In total, we annotated over 6.2 million cells with the islet panel. Of the 975,313 classified islet cells, 328,225 were β-cells.

Using these cell annotations, we analysed β-cells along T1D disease stages. As expected, there was a gradual loss of β-cells from pancreatic islets with advancing disease stage^1^ (Fig. 1e). In non-diabetic donors, β-cells constituted on average 50% of islet cells, whereas donors with long-standing T1D had almost none (Fig. 1e). We found considerable inter-donor variation in β-cell loss within the mAAb+ and Onset T1D groups. Loss of β-cells was accompanied by an expected increase in the α-cell fractions (Extended Data Fig. 3b) and a potential loss in ε-cells in Onset donors. γ- and δ-cells did not show major changes in abundance across disease stages (Extended Data Fig. 3b).

Analysis of key β-cell lineage and functional markers in disease stages before LD T1D (due to the near-total loss of these cells in LD donors) revealed lower levels of several β-cell and islet-lineage markers in later disease stages (Fig. 1f and Extended Data Fig. 3c). This trend was most pronounced in β-cell hormones, with significantly lower levels (*P* < 0.03) of IAPP, ProINS and C-peptide in Onset T1D cases compared with controls (Extended Data Fig. 3c and Fig. 1f; we note that ProINS and C-peptide were measured with separate antibodies). We also detected significantly lower IAPP expression at the mAAb+ stage compared with controls (*P* = 4 × 10^−3^) and sAAb+ donors (*P* = 4.6 × 10^−2^), implicating IAPP as a major marker of early disease progression. HLA-ABC trended higher at the mAAb+ stage compared with the controls (*P* = 0.08), and we observed significant hyperexpression of HLA-ABC in Onset T1D donors compared with control and mAAb+ donors (*P* < 10^−4^) (Fig. 1f). Contrary to expectations^20–22^, we did not observe significantly higher levels of ER-stress markers (IRE1α-P, WFS-1 or XBP1), inflammation or redox markers (NF-κB, CD155 or TXNIP) or IFN-responsive markers (MX1 or ADAR) across disease stages (Extended Data Fig. 3d) and the TIGIT/CD226 receptor CD155 as well as the lowly expressed redox signalling marker TXNIP were instead expressed at significantly lower levels in Onset donors^29,30^ (Extended Data Fig. 3d). Only ADAR tended towards higher levels in Onset donors, indicating an IFN-response in clinical disease (Extended Data Fig. 3d). Indeed, reanalysis of a public scRNA sequencing dataset^31^ showed higher IFN-responses but no heightened ER-stress marker levels (GO_BP: 0030968) or ER-stress-associated TF activity in β-cells from T1D donors compared with controls (Supplementary Fig. 2); of the five stress-associated TFs we could probe in this analysis, XBP1 showed reduced activity in T1D. In total, 24 out of 28 markers had lower protein levels in Onset than in control donors. These data suggest that the functions of remaining β-cells degrade as T1D progresses, including in clinically targeted pathways such as TXNIP.

Most covariates were not associated with β-cell marker expression (Supplementary Fig. 3a,b); this included cause of death and organ transit time, which were unbalanced across disease stages, indicating that these covariates did not influence stage-specific results. β-cell marker expression was associated only with age, with complement component (C3) being higher in younger versus older subjects; however, C3 was not associated with disease stages (Supplementary Fig. 3c,d). Association of β-cell marker expression to age was also limited to Onset donors (Extended Data Fig. 3e). Finally, we tested whether our measured β-cell expression patterns were linked to clinically measured GAD autoantibody (GADA) titres, focusing this analysis on sAAb+ GADA+ donors (*N* = 25). We detected mild inverse associations for stress or ER-stress markers TXNIP (*P* = 2 × 10^−3^), NF-κB (*P* = 0.12) and XBP1 (*P* = 0.12) but not for HLA-ABC (*P* = 0.92) or β-cell lineage markers (*P* > 0.41) (Supplementary Fig. 4). We did not include later disease stages in this analysis as their autoantibody profiles were diverse (Extended Data Fig. 1a).

Different islets from an individual may progress through T1D at different rates, potentially obscuring patterns of disease progression. Therefore, we inferred the temporal sequence of changes in β-cells during disease progression using slingshot pseudotime analysis^32^. Slingshot detected a single lineage (Fig. 1g), which started at control donors, progressed with sAAb+ and mAAb+ as mixed samples between the two endpoints and ended with Onset T1D donors (Extended Data Fig. 3f,g). More specifically, it ended in β-cells in insulitic islets, that is immune infiltrated islets with at least six T cells immediately adjacent to or within the islets^33^ (Extended Data Fig. 3h). The average β-cell pseudotime per donor thus increased significantly over consecutive disease stages (Fig. 1h) and was well correlated to known features of T1D progression such as donor HbA1c status (*r* = 0.64, *P* = 10^−7^), β-cell fraction (*r* = −0.74, *P* = 5 × 10^−14^) and disease stage (*r* = 0.75, *P* = 2 × 10^−14^), confirming the biological relevance of the inferred trajectory.

As in our cross-sectional analysis, analysis of β-cell marker levels along pseudotime showed trends towards lower expression levels in lineage β-cell markers such as PDX1 and NKX6.1 but no significant changes in ER stress, inflammation or IFN-responsive markers (Fig. 1i). We observed lower levels of IAPP and higher levels of HLA-ABC with increasing pseudotime (Fig. 1i and Extended Data Fig. 3i, both *P* < 10^−16^). Lower levels of ProINS and INS were on average observed only in Onset cases, whereas IAPP was significantly less expressed in some mAAb+ islets, which apparently were IAPP-INS+ (Fig. 1f,i,j and Extended Data Fig. 3i). Our data therefore suggest that ProINS and INS loss is preceded by loss of IAPP, despite their known co-secretion^34^.

In summary, starting in the mAAb+ stage, we observed gradually lower expression levels of β-cell lineage markers in comparison with prior disease stages. Levels of IAPP were strongly lower and levels of HLA-ABC were progressively higher across pseudotime, with loss of β-cell hormones before β-cell death. We observed lower levels of 24 out of 28 measured β-cell markers in Onset donors compared with controls, including mildly lower levels of ER-stress and other functional markers, suggesting degradation of β-cell function along disease progression.

### MHC-I, MHC-II and other IFN-response markers are upregulated in islet cells alongside immune cell infiltration

Islet immune infiltration as well as β-cell MHC-I hyperexpression are main hallmarks of T1D^3^. A controversial question has been whether MHC-II is hyper-expressed on β-cells during T1D development^18,35^, given the potential implications for direct antigen presentation from β-cells to CD4^+^ T cells and that initial MHC-II observations on β-cells could not be reproduced. MHC-II hyperexpression was recently reported in pancreatic ductal cells^24^ and β-cells^36,37^ of patients with T1D. Highly multiplexed imaging techniques are needed to establish HLA-DR (MHC-II) expression not only in β-cells but also other islet cells and further to exclude spillover or false annotation. Using our extensive immune panel, we therefore probed for MHC-II hyperexpression in our cohort and tested for co-expression with MHC-I.

**Fig. 2:**
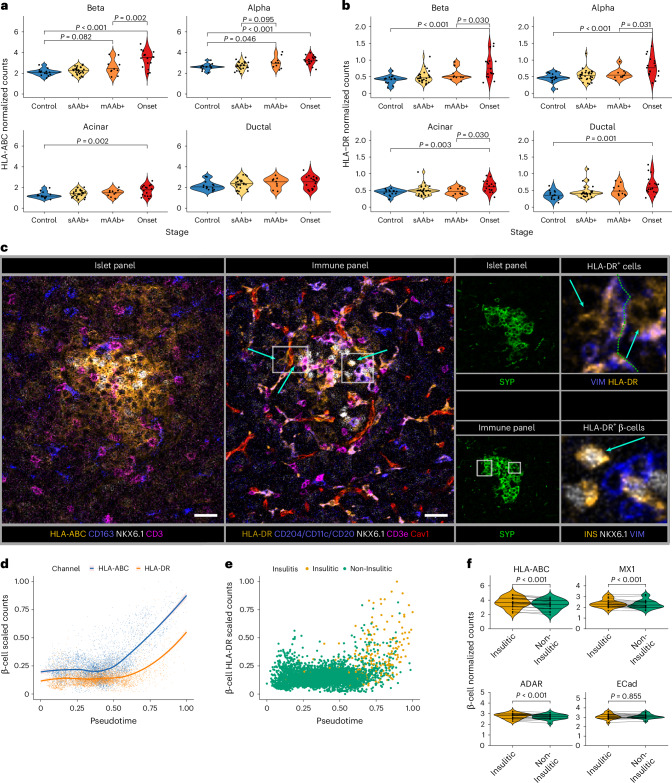
IFN-dependent MHC-II upregulation in β-cells and other pancreatic cells. **a**,**b**, Violin plots of HLA-ABC (**a**) and HLA-DR (**b**) expression across cell types and disease stages (each dot shows mean per donor across ROIs). HLA-ABC and HLA-DR were measured using the islet and immune panel, respectively. The centre line indicates the median. **c**, Representative images of two consecutive sections showing HLA-ABC^+^ cells (left; islet panel) and HLA-DR^+^ cells (centre; immune panel). The arrows indicate HLA-DR^+^ β-cells (NKX6.1+, APC marker−; orange and white) or other HLA-DR^+^ endocrine and exocrine cells (orange). Right: magnified insets correspond to white boxes. The arrow in the magnified upper right panel indicates HLA-DR^+^ endocrine and exocrine cells (orange), both negative for VIM (blue; mesenchymal marker). The green line is the islet edge. Centre-right: low-magnification SYP^+^ islets are shown for comparisons. The arrow in the lower right inset indicates INS^+^/NKX6.1^+^/VIM- β-cells. Scale bar, 75 µm. **d**, HLA-ABC and HLA-DR expression in β-cells plotted over pseudotime (each dot shows mean per ROI). Lines indicate locally weighted scatterplot smoothing (LOWESS) fits with 95% confidence intervals (CIs). **e**, HLA-DR expression in β-cells along pseudotime (each dot shows mean per ROI; colour indicates insulitic and non-insulitic ROIs). **f**, Violin plots comparing β-cell expression in ROIs with (*i* = 172) or without (*i* = 533) insulitic islets from the same Onset donors (*N* = 17). The dots represent donor means; lines connect insulitic and non-insulitic ROIs from the same donor. The violin lines indicate the 25th, 50th and 75th percentiles. Tests: non-significant tests are not shown. Statistical comparisons were performed using linear mixed-effects models (two-sided Wald *t*-tests with Satterthwaite approximation). Significances are FDR adjusted. For **a** and **b**: controls: *N* = 15; sAAb+: *N* = 28; mAAb+: *N* = 10; Onset: *N* = 18.

**Extended Data Fig. 4:**
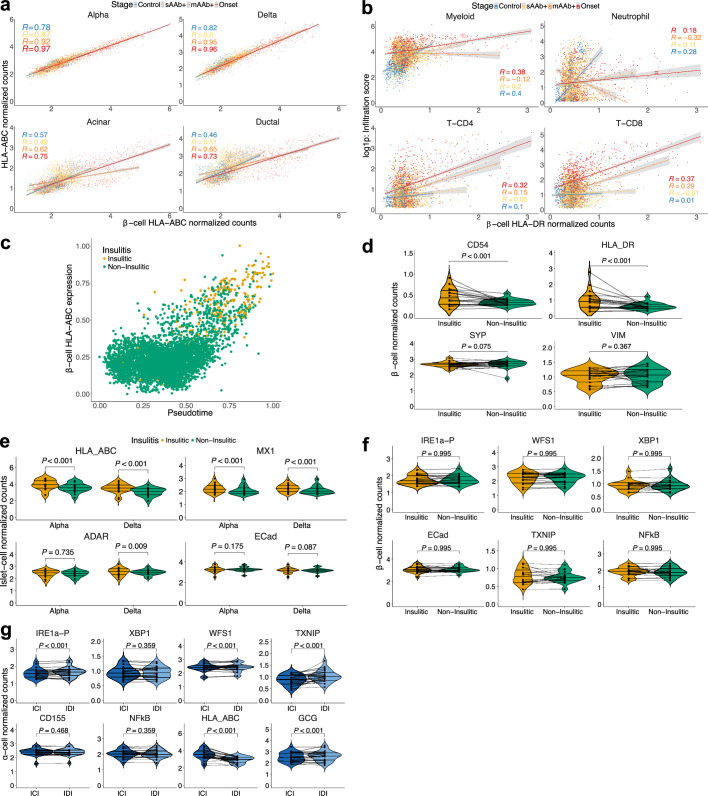
HLA-DR is upregulated in insulitic islets. **a, b,** Correlation of HLA-ABC expression in indicated cell types with expression in β-cells (**a**), and of infiltration score with β-cell HLA-DR expression in the indicated immune cell types (**b**), in both cases across disease stage (each dot shows ROI means). Shown are linear fits with 95% CI; *R*, Spearman correlation coefficient. **c,** β-cell HLA-ABC expression along pseudotime (each dot shows scaled ROI means). **d, e, f**, Violin plots of (**d, f**) β-cell marker expression or (**e**) α-and δ-cell expression comparing ROIs containing insulitic and non-insulitic islets of the same donor (lines connect pairs from the same donor; each dot shows the mean per sample). The same ROIs were analysed as in Fig. 2f. **g,** Violin plots of α-cell marker expression between ROIs with β-cells (ICIs, i = 796 ROIs) versus without β-cells (IDIs, i = 889 ROIs) from Onset donors (N = 21; lines connect pairs from the same donor). Tests: (**d-g**) Linear mixed effects models (two-sided Wald *t*-test with Satterthwaite approximation). Significances are FDR adjusted. Lines show the 25^th^, 50^th^, and 75^th^ percentiles.

We observed higher single-cell expression of both HLA-ABC (MHC-I) and HLA-DR (MHC-II) in multiple endocrine and exocrine cell types across T1D stages with significantly higher expression in Onset T1D relative to control donors and with stronger differences in HLA-ABC than HLA-DR (Fig. 2a–c). Notably, there was also significantly higher MHC-I expression in α-cells in mAAb+ donors, demonstrating that islet-cell perturbations extend to non β-cells before clinical onset (Fig. 2a). There was significant co-expression of HLA-ABC in various islet cell types (for α- and β-cells, Spearman *r* = 0.78–0.97, *P* < 10^−16^; for β-cells and acinar cells, *r* = 0.49-0.75, *P* < 10^−16^), and co-expression increased with disease stage (Extended Data Fig. 4a). This suggests that a pro-inflammatory microenvironment, probably IFN-driven^7^, upregulates islet and islet-proximal HLA-ABC protein levels. In islets, antigen-presenting cells (APCs) and inflamed endothelium expressed HLA-DR at high levels, but we also clearly detected HLA-DR^+^ β-cells without potential spillover from major APC, phagocyte or endothelial markers in inflamed islets (for example, VIM, CD204, CD20, CD11c and CAV1) (Fig. 2c). Further, we observed other HLA-DR^+^ endocrine and exocrine cells, at lower HLA-DR intensity than in β-cells (Fig. 2b,c). To determine the (pseudo)temporal sequence of changing HLA-DR and HLA-ABC levels, we aligned expression of these markers in β-cells along pseudotime. This predicted that HLA-ABC upregulation in these cells occurs before upregulation of HLA-DR (Fig. 2d).

More specifically, higher HLA-DR expression in β-cells was correlated with abundance of myeloid and especially T cells (Extended Data Fig. 4b and Supplementary Fig. 5) and was mostly confined to insulitic islets (Fig. 2e). By contrast, higher HLA-ABC levels were not confined to insulitic islets (Extended Data Fig. 4c). β-cells (and other islet cells) in insulitic islets had higher levels not only of HLA-DR and HLA-ABC relative to paired non-insulitic islets of the same donor but also of IFN-responsive markers MX1, ADAR and CD54 (Fig. 2f and Extended Data Fig. 4d,e). In sum, this suggests an IFN-driven β-cell and islet cell state specifically associated with T cell infiltration and marked by HLA-DR expression.

We detected no significant differences in ER stress markers of β-cells between paired insulitic and non-insulitic islets of the same donor (Extended Data Fig. 4f). Furthermore, comparison of α-cells of islets with β-cells (ICIs) to those without β-cells (IDIs) of the same Onset donors showed that α-cells in ICIs showed higher HLA-ABC levels, as expected under more inflammatory conditions, but lower ER-stress marker levels (IREα-P and WFS-1) (Extended Data Fig. 4g). In both these analyses, we thus, surprisingly, did not detect higher activity of our measured ER-stress markers under conditions of islet inflammation.

In sum, these data suggest that in islets, MHC-I is upregulated first, coinciding with protein downregulation in most lineage and functional β-cell markers, followed by upregulation of MHC-II and other IFN-responsive markers during T cell infiltration. Importantly, these changes were observed not only in β-cells but also in other islet and islet-proximal endocrine and exocrine cells.

### Immune cell types change in density during disease progression

**Fig. 3:**
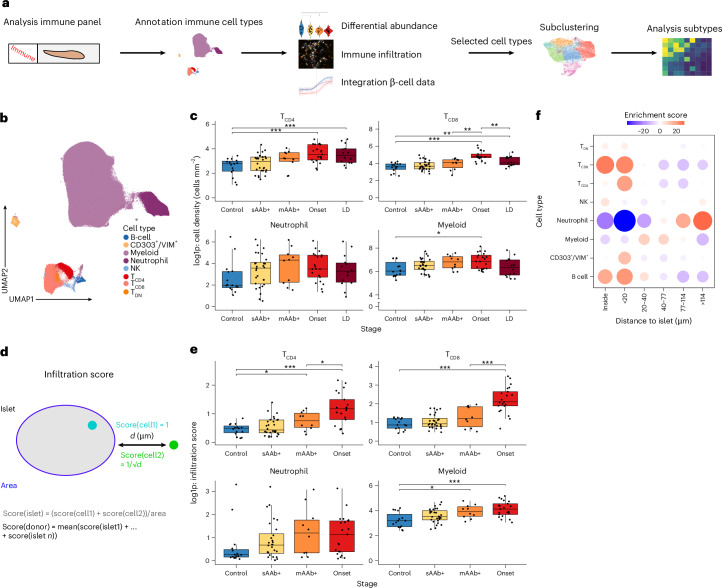
Immune cell type dynamics across T1D disease stages. **a**, Data analysis with the immune panel. **b**, Uniform manifold approximation and projection of immune cell expression profiles, coloured by cell type. **c**, Mean densities of indicated cell types across disease stages (each dot shows mean per donor across ROIs). The centre line indicates the median, box bounds the interquartile range (IQR) and whiskers extend to 1.5× IQR. The Myeloid cell facet contains a *y*-axis break. **d**, Sketch of the developed infiltration score, normalized by islet size. **e**, Mean infiltration scores of the indicated cell types across disease stages (each dot, shows mean per donor across ROIs containing β-cells (ICIs)). Boxplots are as in **c**. **f**, Enrichment of immune cell types by distance to the islet edge (score: *χ*^2^ residuals; red, enrichment; blue, depletion). Tests: *P < 0.05; **P < 0.01; ***P < 0.001 for all comparisons. Non-significant tests are not shown. Differential abundance was determined using edgeR (two-sided empirical Bayes quasi-likelihood *F*-tests) and differential infiltration scores using a linear mixed-effects model (two-sided Wald *t*-test with Satterthwaite approximation). Significances are FDR adjusted. For **c** and **e**: controls: *N* = 15; sAAb+: *N* = 28; mAAb+: *N* = 10; Onset: *N* = 21. For **c:** LD: *N* = 14. The transformation log1p is short for log_e_(1 + *x*), with *x* being the cell density or infiltration score, depending on the subpanel.

**Extended Data Fig. 5:**
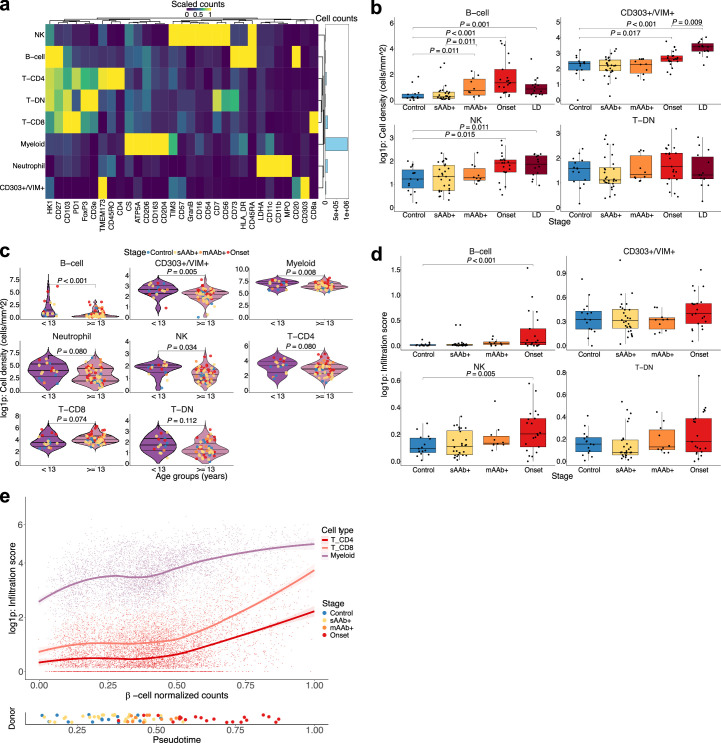
Immune cells infiltrate islets during disease progression. **a**, Lineage marker expression across annotated immune cell types; barplot indicates total cell counts. **b**, Mean densities of indicated cell types across disease stages (each dot, sample means across ROIs). Center line indicates the median, box bounds represent the IQR, and the whiskers extend to 1.5x the IQR. **c**, Cell type densities by donor age group (each dot, sample means across ROIs; coloured by disease stage). Lines are the 25th, 50th, and 75th percentiles. **d**, Infiltration scores across disease stages (each dot, donor means across ROIs containing beta-cells (ICIs)). Boxplots are as in (**b**). **e**, Immune cell infiltration scores for the indicated cell types along pseudotime (dots and fits coloured by cell type; shaded areas, 95 % CI). Tests: Non-significant tests are not shown. (**b**, **c**) Differential abundances were calculated using edgeR (two-sided empirical Bayes quasi-likelihood F-test) and (**d**) differential infiltration scores by linear mixed effects models (two-sided Wald t-test with Satterthwaite approximation). Significances are FDR adjusted. (**b**, **c**, **d**): Controls: *N*=15; sAAb+: *N*=28; mAAb+: *N*=10; Onset: *N*=21; LD: (**b**) *N*=14.

To study the evolution of the immune compartment within and proximal to islets during T1D progression, we used the immune panel data (Fig. 3a). We first separated cells into immune and non-immune cell types and classified non-immune cell types as acinar, ductal, mesenchymal, nerves, endothelial, smooth-muscle or ‘other’. Next, immune cells were annotated as myeloid cells, neutrophils, natural killer (NK) cells, B cells, CD4^+^ helper T (T_CD4_) cells, CD8^+^ cytotoxic T (T_CD8_) cells, double-negative T (T_DN_) cells (CD3e^+^CD4^−^CD8^−^, which probably include NK T cells and γδ T cells) and CD303^+^/VIM^+^ cells (Fig. 3b and Extended Data Fig. 5a). CD303^+^/VIM^+^ cells are probably a fibroblast subset involved in the fibrotic process after β-cell destruction or potentially an atypical plasmacytoid DC (CD303^+^HLA-DR^−^) (Extended Data Fig. 5a). In total, about 10.5 million cells were annotated, and approximately 1.2 million were immune cells (Fig. 3b).

We quantified immune cell density along T1D progression to identify disease-relevant immune cell types. Donor age and time in ICU but no other covariates were associated with immune cell type densities, and we thus adjusted for these covariates in all immune cell analyses (Methods; Supplementary Fig. 6). Myeloid, B, NK and T cells displayed gradually higher densities with stage, beginning in early disease stages, with significantly higher densities over control samples in Onset donors for all these immune cell types (Fig. 3c and Extended Data Fig. 5b). B cell density was variable between donors, with significantly higher fractions in younger T1D donors than in those ≥13 years of age (Extended Data Fig. 5c), as expected^38^. Neutrophil densities were variable, with outlier cases within each group showing considerable neutrophil presence (Fig. 3c).

Both increase in abundance of immune cells within the pancreas and attraction to islets are probably relevant for T1D progression. Therefore, we developed an ‘infiltration score’ that combines distance to islet edge and cell abundance in the measured ROIs and normalizes for islet size (Fig. 3d). We observed a significant increase in infiltration score for T cells, B, NK and myeloid cells in Onset T1D and some mAAb+ donors, compared with controls (Fig. 3e and Extended Data Fig. 5d). Both T_CD4_ and T_CD8_ cells also showed increased infiltration along pseudotime (*r* = 0.78, *P* < 10^−16^), suggesting co-infiltration of these cell types (Extended Data Fig. 5e). Further, infiltration score changes over pseudotime suggest that myeloid cells potentially precede T cells (*P* = 0.18) (Extended Data Fig. 5e). Other cell types showed no changes in infiltration score along T1D disease stages, with neutrophils displaying specific islet avoidance (Fig. 3e,f, Extended Data Fig. 5d and Supplementary Fig. 7a). Based on these results, we focused on the major islet-infiltrating immune cells, that is T_CD4_, T_CD8_ and myeloid cells.

### PD1^+^CD4^+^ T cells and exhausted-like CD8^+^ T cells are enriched in early T1D islet inflammation

T cell-targeting therapies have shown partial therapeutic success before and after clinical onset of T1D^7^. However, while T cell states have been comprehensively evaluated in peripheral blood, they remain incompletely characterized in human pancreata, especially from patients who are AAb+. We thus analysed potential T_CD4_ and T_CD8_ states in our cohort.

**Fig. 4:**
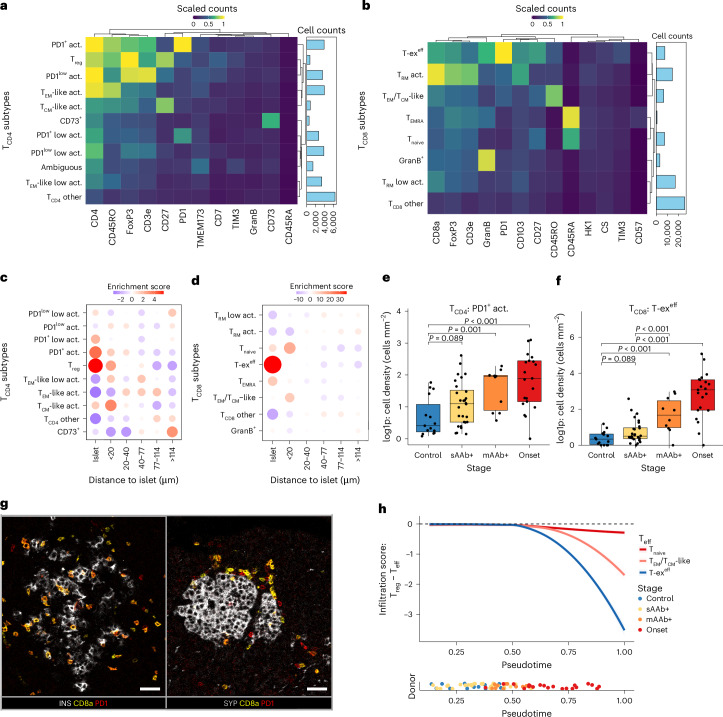
Exhausted-like T cells dominate islet infiltration in mAAb+ and T1D donors. **a**,**b**, Lineage marker expression across annotated T_CD4_ (**a**) and T_CD8_ (**b**) subtypes. Right: barplots indicate total cell counts. **c**,**d**, Enrichment scores of T_CD4_ (**c**) and T_CD8_ (**d**) cell subtypes by distance to the islet edge (score: *χ*^2^ residuals; red, enrichment; blue, depletion). **e**,**f**, Mean densities of PD1^+^ act. cells (**e**) and T-ex^eff^ cells (**f**) across disease stages (each dot shows mean per donor across ROIs containing β-cells (ICIs)). The centre line indicates the median, box bounds the interquartile range (IQR) and whiskers extend to 1.5× IQR. The transformation log1p is short for log_e_(1 + *x*), with *x* being the cell density. **g**, Representative images of islet (left) and peri-islet enrichment (right) of exhausted-like T cells from an Onset donor and mAAb+ donor, respectively. Scale bar, 75 µm. **h**, Infiltration scores of T_CD8_ effector cells (T_eff_) relative to T_reg_ along pseudotime per ROI. Negative values indicate higher presence of T_eff_ than T_reg_ cells in islets. Lines indicate LOWESS fits. Bottom: donor-average pseudotime is shown. Tests: non-significant tests are not shown. Differential abundance was tested using edgeR (two-sided empirical Bayes quasi-likelihood *F*-tests). Significances are FDR adjusted. For **e** and **f**: controls: *N* = 15; sAAb+: *N* = 28; mAAb+: *N* = 10; Onset: *N* = 21.

We assigned around 27,000 T_CD4_ cells to eleven T_CD4_ subtypes (Fig. 4a). These included activated and minimally activated CD45RO^+^CD45RA^−^CD27^−^ effector memory-like cells (T_EM_-like activated (act.), T_EM_-like low act.), CD45RO^+^CD45RA^−^CD27^+^ central memory-like cells (T_CM_-like act.), PD1^+^T_CD4_ memory cells (PD1^+^ act., PD1^+^ low act.) and PD1^low^ cells (PD1^low^ act., PD1^low^ low act.), as well as two regulatory phenotypes, CD73^+^ T_CD4_ cells and regulatory T cells (T_reg_). We also labelled T_CD4_ cells with ambiguous expression profiles and an undefined T_CD4_ cluster (T_CD4_ other). Next, we assigned around 72,000 T_CD8_ cells to eight T_CD8_ cell subtypes (Fig. 4b): CD45RA^+^CD27^+^CD57^−^ naive T_CD8_ cells, CD45RA^+^CD27^low^CD57^+^ effector memory cells re-expressing CD45RA T cells (T_EMRA_), activated and marginally activated CD45RO^+^CD103^+^ tissue-resident memory cells (T_RM_ act., T_RM_ low act.), CD45RO^+^CD27^mid^ effector memory/central memory-like cells (T_EM_/T_CM_-like), GranB^+^ cytotoxic T_CD8_ cells, an undefined set of T_CD8_ cells (T_CD8_ other) and a T_CD8_ memory cell subtype that expressed high levels of markers of exhaustion (PD1 and TIM-3), cytotoxicity (GranB), survival (CD27) and metabolism (for example, citrate synthase (CS)). This suggested an exhausted-like phenotype with some cytotoxic effector functions (T-ex^eff^), although we note that TOX and TCF1 were not measured in our panels.

**Extended Data Fig. 6:**
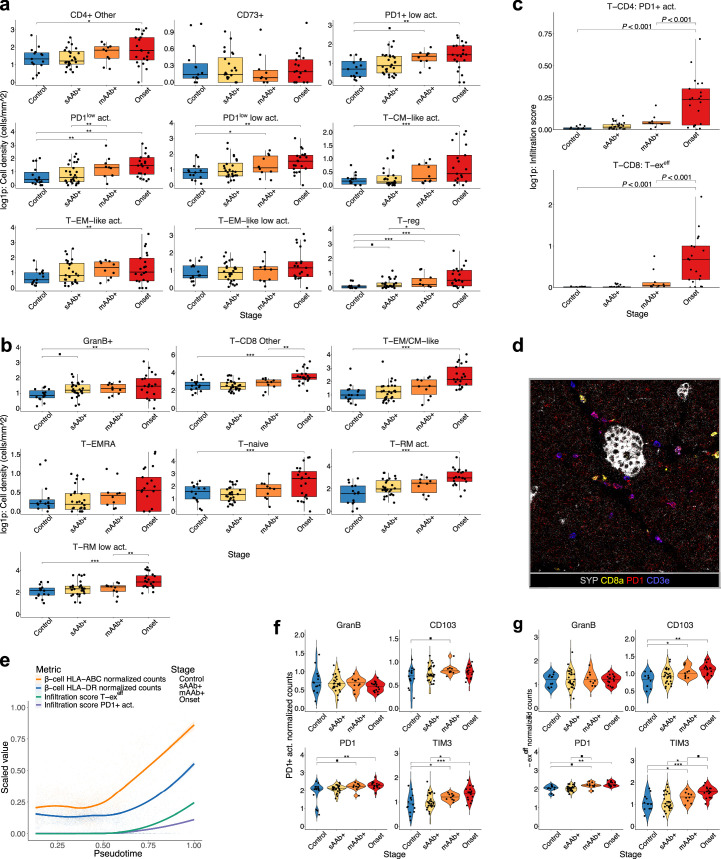
Cell density and infiltration scores of T-CD4 and T-CD8 cell subtypes. **a, b,** Mean densities of T-CD4 **(a)** and T-CD8 **(b)** cell subtypes across disease stages (each dot, mean per donor across ROIs containing β-cells (ICIs)). The center line indicates the median, box bounds the IQR, and whiskers extend to 1.5x the IQR. **c,** Mean infiltration scores of PD1+ act. cells (upper) and T-ex^eff^ cells (lower) by disease stage (each dot, mean per donor across ICIs). Box plots as in **(a, b)**. **d,** Image shows PD1+ act. cells (CD3e^+^PD1^+^; purple) in islet-proximal exocrine tissue, but not islets (SYP+, gray) in sAAb+ donors. **e,** Scaled infiltration scores of PD1+ act. cells and T-ex^eff^ cells, and min-maxed β-cell HLA-ABC and HLA-DR expression values plotted along pseudotime. Data shows mean per ROI. Shown are LOWESS fits with 95 % CI. **f, g,** Expression levels of the indicated markers in PD1+ act. cells **(f)** and T-ex^eff^ cells **(g)** across disease stages (each dot shows mean per donor across ICIs). The center line indicates the median. Tests: • *P* < 0.1; ^*∗*^ *P* < 0.05; ^∗∗^ *P* < 0.01; ^∗∗∗^ *P* < 0.001 for all comparisons. Non-significant tests are not shown. **(a, b)** Adjusted significances were determined for each stage by testing for differential abundance using edgeR (two-sided empirical Bayes quasi-likelihood *F*-test). **(c, f, g)** Significances were computed by fitting a linear mixed-effects model (two-tailed Wald *t*-test with Satterthwaite approximation). **(a-c, f):** controls: N = 15; sAAb + : N = 28; mAAb+: N = 10; Onset: (**a-c**) N = 21 or (**f, g**) N = 17.

Next, we analysed islet-enrichment and abundance of T cell subtypes across progression. For T_CD4_ cells, we observed islet-enrichment of T_reg_ (*P* = 5 × 10^−7^) and PD1^+^ act. cells (*P* = 4 × 10^−4^) (Fig. 4c)—and for T_CD8_ cells—of T-ex^eff^ cells (*P* < 10^−16^) (Fig. 4d), especially in Onset donors (Supplementary Fig. 7b,c). The islet-enriched PD1^+^ subtypes (that is, T-ex^eff^ and PD1^+^ act. cells) and T_reg_ demonstrated significantly greater abundance in mAAb+ and Onset donors compared with controls (all *P* < 2 × 10^−3^) (Fig. 4e,f and Extended Data Fig. 6a) and a tendency towards higher abundance in sAAb+ donors (all *P* < 0.1). Also enriched in mAAb+ samples were PD1^low^ act., and PD1^low^ low act. cells (Extended Data Fig. 6a,b), but these were not enriched in islets (Fig. 4c), suggesting limited cytotoxic effects at this stage of disease.

Using our infiltration score, we found that PD1^+^ act. and T-ex^eff^ cells significantly infiltrated islets in Onset donors and a subset of mAAb+ donors but not in sAAb+ donors compared with controls (Extended Data Fig. 6c). In sAAb+ donors, PD1^+^ act. cells were mostly confined to the islet-proximal pancreas (Extended Data Fig. 6d). Notably, infiltration scores of PD1^+^ act. and T-ex^eff^ cells subtypes displayed the highest association to β-cell MHC-I and MHC-II expression of all T cell subtypes along pseudotime (all *r* > 0.72, *P* < 10^−14^), with infiltration following MHC-I expression and coinciding with MHC-II expression on β-cells (Extended Data Fig. 6e). With disease progression, exhaustion-markers PD1 and TIM-3 were more strongly expressed for both cell subtypes, as was the tissue-residency marker CD103 for T-ex^eff^ cells, which might also signal exhaustion^39^; the cytotoxicity marker GranB did not change (Extended Data Fig. 6f,g). Together these data suggest exhaustion of these cell subtypes within the islet microenvironment.

**Extended Data Fig. 7:**
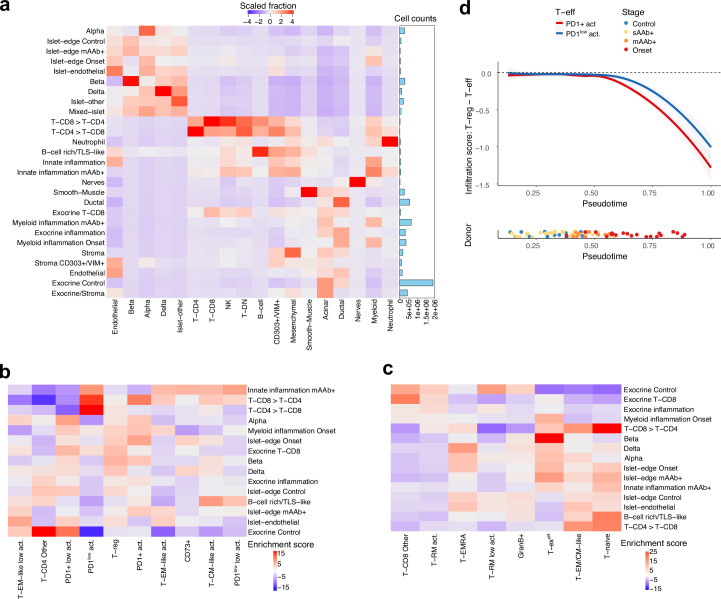
Enrichment of exhausted-like T cells in peri-islet and β-cell cellular neighborhoods. **a,** Cell type composition of aggregated cellular neighborhoods (CNs). Columns are scaled by cell type fractions in the indicated CNs. Red indicates higher fractions, blue lower fractions. Barplot to the right indicates total cells counts. **b, c,** Enrichment of **(b)** T-CD4 and (**c**) T-CD8 subtypes in informative CNs (matrix values: χ^2^ residuals; red, enrichment; blue, depletion). **d,** Infiltration scores of T-CD4 effector cells (T-eff) (PD1+ act., PD1^low^ act.) along pseudotime per ROI, subtracted against T-reg infiltration scores. Negative values thus indicate higher islet presence of T-eff than T-reg cells. LOWESS fits with 95 % CI. Donor-averaged pseudotime is shown in lower panel.

Next, we analysed the spatial niches of T cell subtypes to determine if these were reflective of diabetogenic processes. We identified the ten nearest neighbours of all cells, clustered the resulting cell populations into 70 cellular neighbourhoods (CNs)^40^ and then manually annotated them by their enriched cell types, localization and disease stage association (Extended Data Fig. 7a). Similar CNs were aggregated to yield 28 CNs. This analysis showed stronger enrichment of PD1^+^ act. cells and T-ex^eff^ cells in β-cell enriched CNs (‘beta’) than in other islet-cell CNs (Extended Data Fig. 7b,c), with especially T-ex^eff^ cells often in direct contact with β-cells (Fig. 4g). We also observed enrichment of both subtypes at the islet edge in mAAb+ and Onset donors (‘islet-edge mAAb+’, ‘islet-edge Onset’), which probably captures peri-islet accumulation at these stages before overt islet infiltration (Fig. 4g and Extended Data Fig. 7b,c).

In Onset donors, most non-exhausted T cell subtypes were enriched in comparison with controls (Extended Data Fig. 6a,b). Both infiltration scores and enrichment in T cell-rich CNs (‘T_CD4_ > T_CD8_’, ‘T_CD8_ > T_CD4_’) suggested key roles for T_CD4_ PD1^low^ act. cells, which are probably early effector cells (Extended Data Fig. 7b), as well as for T_naive_ and T_EM_/T_CM_-like cells (Extended Data Fig. 7c). These non-exhausted T cell subtypes appeared to follow accumulation of exhausted-like T cells into islets along pseudotime, apparently outcompeting T_reg_ in islets of Onset donors (Fig. 4h and Extended Data Fig. 7d).

In sum, this indicates PD1^+^ T cell subtypes as critical indicators of early disease, with progressive shifts from islet-proximal tissue sites in AAb+ donors, to overt islet infiltration in Onset donors. Their β-cell directed infiltration is strongly linked to islet inflammation, especially MHC-II expression levels. Although the islet microenvironment appears to limit cytotoxicity of these cells by further exhaustion upon infiltration, with potentially also T_reg_ playing a role, expression profiles of T-ex^eff^ cells suggest remaining cytotoxicity and thus contribution to β-cell demise.

### Peri-islet macrophages are M1 polarized

Other than T cells, myeloid cells are the main immune cells that infiltrate islets during T1D progression (Fig. 3). Pancreatic myeloid cells were shown to be important for T1D pathogenesis in mouse models^13^, but their role has been scarcely studied in humans^16^. We therefore analysed myeloid cells over disease progression.

**Fig. 5:**
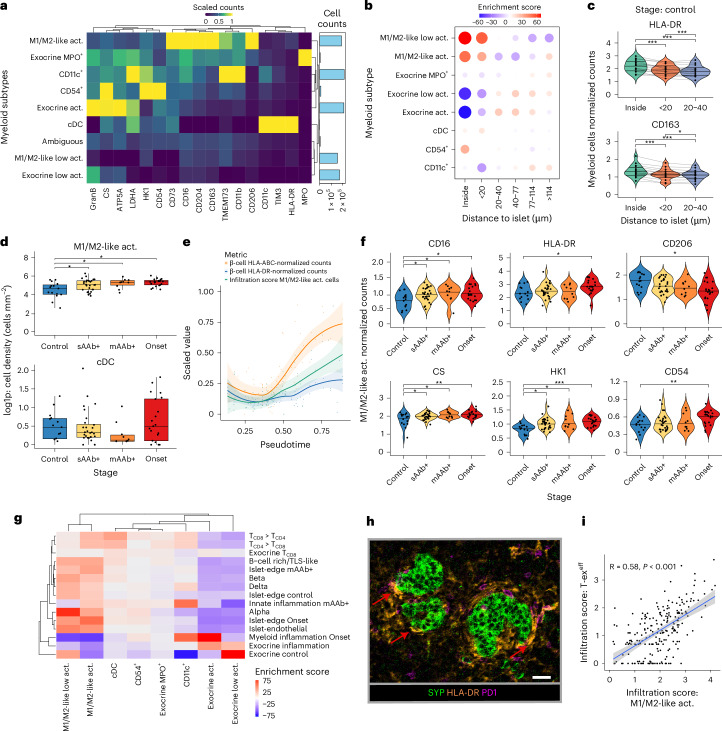
Activated M1/M2-like macrophages and cDCs interact with lymphocytes within and near islets. **a**, Lineage marker expression across annotated myeloid cell subtypes; barplots (right) indicate total cell counts. **b**, Enrichment of myeloid cell subtypes by distance to the islet edge (score: *χ*^2^ residuals; red, enrichment; blue, depletion). **c**, Expression of indicated markers in islet and peri-islet myeloid cells from 15 non-diabetic donors (each dot shows mean per donor across ROIs). Lines connect paired data by donor. Violin plot lines indicate the 25th, 50th and 75th percentile. **d**, Densities of indicated myeloid cell subtypes across disease stages (each dot shows mean per donor across ROIs). The centre line indicates the median, box bounds the interquartile range (IQR) and the whiskers extend to 1.5× the IQR. The transformation log1p is short for log_e_(1 + *x*), with *x* being the cell density. **e**, Scaled HLA-ABC and HLA-DR β-cell expression and infiltration scores of M1/M2-like act. macrophages along β-cell pseudotime (each dot shows mean per donor); lines indicate LOWESS fits with 95% CIs. **f**, Levels of indicated markers in activated M1/M2 macrophages across disease stages (each dot, mean per donor across ROIs containing β-cells). The centre line is the median. **g**, Enrichment of myeloid subtypes in CNs (scores: *χ*^2^ residuals; red, enrichment; blue, depletion). **h**, Representative ROI from a mAAb+ donor with substantial peri-islet immune infiltration. Red arrows indicate HLA-DR^high^ cDCs (orange) at the SYP^+^ islet edge (green) in close contact with PD1^+^ T cells (magenta). Scale bar, 75 µm. **i**, Unnormalized infiltration scores of T-ex^eff^ cells and M1/M2-like act. cells in insulitic islets (containing β-cells) (*i* = 221). Line indicates a linear fit with 95% CIs. Two-sided Spearman’s rank correlation was used to test association. Tests: *P < 0.05; **P < 0.01; ***P < 0.001 for all comparisons. Non-significant tests are not shown; significances are FDR adjusted. Differential abundance was tested using edgeR (two-sided empirical Bayes quasi-likelihood *F*-test) and differential expression using a linear mixed-effects model (two-sided Wald *t*-test with Satterthwaite approximation). For **d** and **f**: controls: *N* = 15; sAAb+: *N* = 28; mAAb+: *N* = 10; Onset: *N* = 21.

We used a combination of lineage marker expression, activation states and spatial location within the pancreas to annotate nine myeloid subtypes among the roughly 800,000 myeloid cells in our dataset (Fig. 5a). These included activated and minimally activated exocrine macrophages (exocrine act., exocrine low act.) and (peri)-islet-enriched M1/M2-like macrophages (M1/M2-like act., M1/M2-like low act.) with expression of both classical M1 and M2 markers (HLA-DR, CD163 and CD206) (Fig. 5a,b). Further, we annotated conventional DCs (cDCs), characterized by high levels of TIM-3, HLA-DR and CD11c and low levels of macrophage markers CD163 and CD206. These cells were rare (~1,100 cells) and have, to our knowledge, not been defined in human multiplexed T1D imaging studies so far. We annotated further clusters as CD54^+^ macrophages, exocrine MPO^+^ macrophages, CD11c^+^ macrophages and cells with non-myeloid-specific expression profiles as ‘ambiguous’.

**Extended Data Fig. 8:**
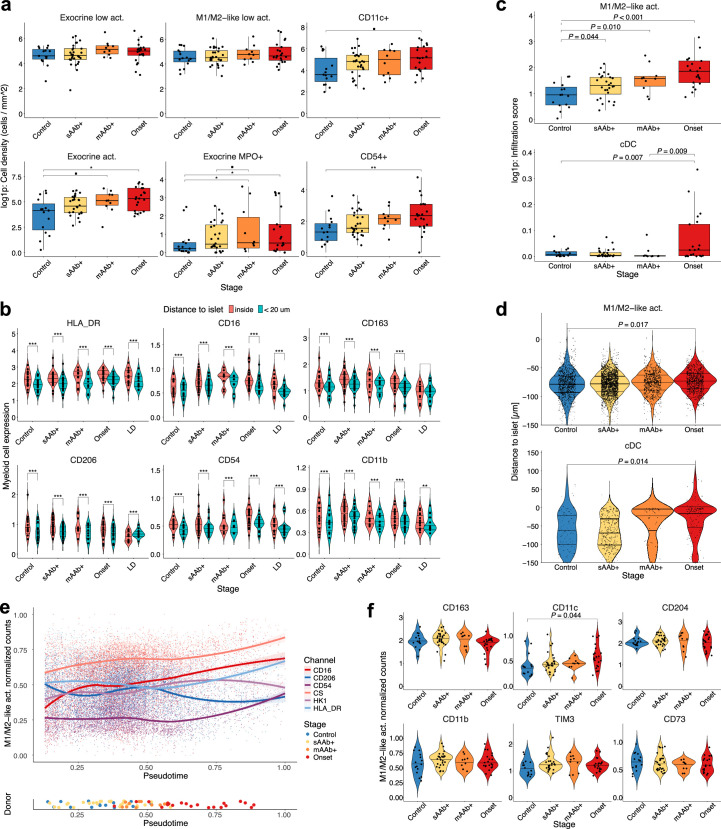
cDCs and activated M1/M2-like macrophages are attracted to islets in the T1D pancreas. **a, c,** Boxplots comparing **(a)** densities and **(c)** infiltration scores of myeloid cell subtypes across disease stage (each dot shows donor means across ROIs containing β-cells (ICIs)). The center line indicates the median, box bounds the IQR, and whiskers extend to 1.5x the IQR. **b,** Expression of key functional and lineage markers between islet versus peri-islet myeloid cells across disease stages (each dot shows donor means across ROIs and distance bins). Lines are the 25^th^, 50^th^, and 75^th^ percentiles. **d,** Distances of M1/M2-like act. macrophages and cDCs to islets across disease stages (cDCs, single-cell distances; macrophages, ROI means due to abundance differences). **e,** Scaled expression levels of key myeloid markers along β-cell pseudotime trajectory. Donor-averaged pseudotime is shown in lower panel. **f,** Expression of myeloid and metabolic markers in M1/M2-like act. macrophages across disease stages (each dot shows donor mean across ICIs). Tests: • *P* < 0.1; ^∗^ *P* < 0.05; ^*∗∗*^ *P* < 0.01; ^*∗∗∗*^ *P* < 0.001 for all comparisons. Non-significant tests are not shown. **(a)** Differential abundance was determined using edgeR (two-sided empirical Bayes quasi-likelihood *F*-test). **(b-d, f)** Differential expression, infiltration scores or distances were computed by linear mixed-effects models (two-sided Wald *t*-test with Satterthwaite approximation). **(a-d, f):** controls: N = 15; sAAb+: N = 28; mAAb+: N = 10; Onset: N = 21. LD: (**b**) N = 14.

Almost all subtypes showed a higher abundance with increasing disease stage (Extended Data Fig. 8a) for example, exocrine MPO^+^, exocrine act. and CD54^+^ macrophages were more abundant in Onset donors than at earlier stages (Extended Data Fig. 8a). Several key myeloid markers (CD163, CD206, CD16, HLA-DR, CD54 and CD11b) were more strongly expressed in islet-resident myeloid cells than in myeloid cells outside islets in all disease stages (Extended Data Fig. 8b), including non-diabetic controls (Fig. 5c). This highlights the importance of immune cell composition and activation within the peri-islet exocrine niche in both homeostasis and T1D progression.

**Extended Data Fig. 9:**
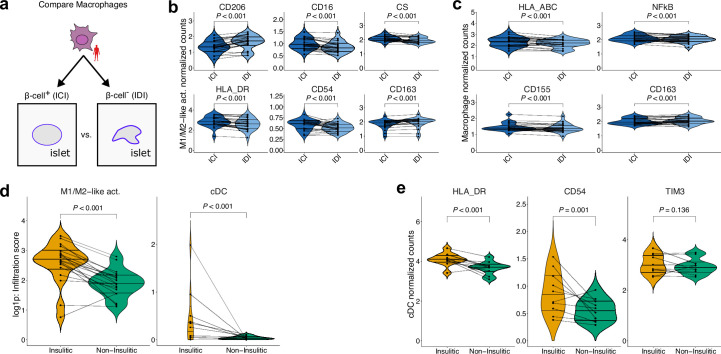
Myeloid cells have pro-inflammatory signatures in insulitic and ICI ROIs. **a,** Schematic of macrophage phenotype comparison between ROIs with β-cells (ICIs) and those without (IDIs) from the same donor. **b, c,** Expression of key myeloid markers for **(b)** M1/M2-like act. macrophages and **(c)** peri-islet and islet macrophages as defined on the Islet panel (<20 µm to the islet edge) comparing ICIs (i = 796) and IDIs (i = 875) of the same Onset donors (N = 21). ICIs and IDIs of the same donors are connected by lines. **d,** Violin plots comparing infiltration scores for indicated cell subtypes in insulitic ROIs (M1/M2-like act.: N = 19 donors, i = 179 ROIs, cDCs: N = 10 donors, i = 148 ROIs) and non-insulitic ROIs (M1/M2-like act.: i = 552 ROIs, cDCs: i = 268 ROIs) of the same donors. Only ICI ROIs of Onset T1D donors are considered; lines connect insulitic and non-insulitic ROIs of the same donor. **e:** Violin plots of cDC marker expression from the same insulitic and non-insulitic ROIs as in **(d)**; ROI pairs per donor are connected by lines. Tests: Differential expression was assessed using a linear mixed-effects model (two-sided Wald *t*-test with Satterthwaite approximation). Significances are FDR adjusted. (**b-e**) Lines are the 25^th^, 50^th^, and 75^th^ percentiles.

Given their roles in peripheral immunity/tolerance, M1/M2-like act. macrophages and cDCs are probably critical in T1D^41^. M1/M2-like act. macrophages tended to be more frequent in the sAAb+ stage compared with controls (*P* < 0.1), with some inter-patient variability (Fig. 5d). By contrast, cDCs did not change frequency over disease progression (Fig. 5d). We observed a shift of both cDCs and M1/M2-like act. macrophages to the islet edge in mAAb+ and Onset (and partly in sAAb+) donors relative to controls (Extended Data Fig. 8c,d and Supplementary Fig. 7d). The M1/M2-like act. macrophage infiltration score followed the same trajectory over pseudotime as HLA-ABC expression in β-cells, especially during initial HLA-ABC upregulation, and showed cross-correlation to HLA-DR in later pseudotime (Fig. 5e). This suggests that infiltration of these macrophages is associated with both early and late islet inflammation. Marker expression in M1/M2-like act. macrophages differed over disease progression, with higher expression of metabolism markers (for example, CS and HK1), IFN-dependent pro-inflammatory markers (CD54 and HLA-DR) and monocyte markers (CD16) with progressing disease course (Fig. 5f). Interestingly, some of these expression changes were observed early in the disease (that is, CS, CD16 and HK1 differed from control donor levels in the sAAb+ stage). We also observed lower expression of classical M2 markers, such as CD206, over the disease course (Fig. 5f and Extended Data Fig. 8e). Differences in levels of other major myeloid markers, besides CD11c, were not significant (Extended Data Fig. 8f). We investigated whether the phenotypes of these M1/M2-like act. macrophages change after the loss of β-cells by comparing islets with (ICI) and without (IDI) β-cells from the same Onset donors. We observed upregulation of classical M2 markers (that is, CD163 and CD206) and downregulation of activation and pro-inflammatory markers (for example, CD54 and HLA-DR) in M1/M2-like act. macrophages from IDIs compared with ICIs (Extended Data Fig. 9a–c). Overall, these data suggest that islet inflammation that occurs early in the disease results in attraction, activation and M1-polarization of islet-resident M1/M2-like act. macrophages. This probably also includes attraction of monocytes. After loss of β-cells, macrophages return to an M2-like phenotype.

Next, we analysed enrichment of myeloid subtypes in CNs. Importantly, both M1/M2-like act. macrophages and cDCs were enriched in T cell-rich CNs (‘T_CD8_ > T_CD4_’ or ‘T_CD4_ > T_CD8_’), suggesting myeloid cell interactions with both T-CD4 and T-CD8 cells (Fig. 5g). M1/M2-like act. macrophages were enriched around islet-endothelial cells and the islet edge, spatially positioning them as key regulators of invading lymphocytes. Interestingly, they were especially enriched at the islet edge in mAAb+ donors, as were exhausted-like T cells (Extended Data Fig. 7b,c), suggesting interactions between these cell types near islets before overt islet infiltration (Fig. 5g). Indeed, we observed cDCs and M1/M2-like act. macrophages in direct contact with T cells, including exhausted-like T cells, at the islet periphery (Fig. 5h). Further, M1/M2-like act. macrophages and cDCs were enriched in insulitic islets versus paired non-insulitic islets of the same donor (Extended Data Fig. 9d). Both subtypes displayed significant upregulation of IFN-linked markers CD54 and HLA-DR in insulitic islets (Extended Data Fig. 9e), which M1/M2-like act. macrophages co-infiltrated with T-ex^eff^ cells (Fig. 5i). This indicates IFN-dependent footprints linked to T-ex^eff^ cells in myeloid cells and enhanced capacity for activation of T_CD4_ cells. By contrast, high TIM-3 levels on cDCs (Fig. 5b), also suggests a regulatory programme that limits autoimmunity in the pancreas^42^. In sum, these data suggest that specific interactions between M1-polarized macrophages and cDCs with T cells, especially exhausted-like T cells, at the islet periphery and within islets can modulate autoimmunity.

### Identification of spatial, progression and age-associated immune cell motifs

**Fig. 6:**
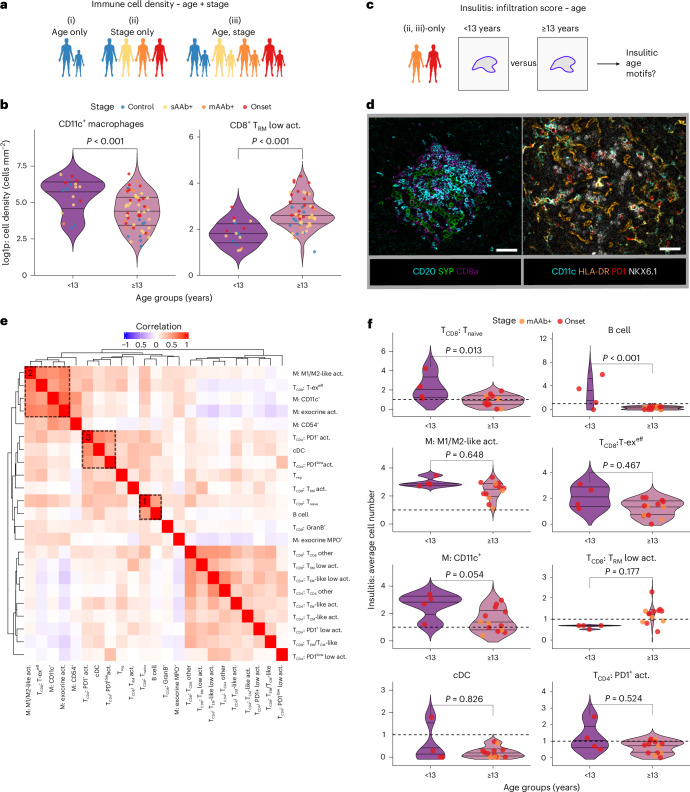
Age-associated immune cell phenotypes form insulitic clusters. **a**, A schematic showing that immune cell subtypes may be (i) age associated, (ii) stage associated or (iii) both age and stage associated. The single tilde (~) denotes a model formula relating cell density to age and stage. **b**, Violin plots of mean density of the indicated cell types in younger (<13 years) versus older (≥13 years) donors (each dot shows mean per donor across ROIs). Additional subtypes are shown in Extended Data Fig. 10a. Lines are the 25th, 50th and 75th percentiles. The transformation log1p is short for log_e_(1 + *x*), with *x* being the cell density. **c**, A comparison of (ii) stage-associated and (iii) age- and stage-associated subtypes between insulitic islets of younger and older donors. **d**, Micrographs showing insulitic immune motifs in Onset donors (<13 years). Left: strong islet infiltration (SYP^+^) of both CD8a^+^ T cells and CD20^+^ B cells (cluster 1 in **e**). Right: insulitic infiltrate comprising CD11c^+^/HLA-DR^+^ macrophages in contact with PD1^+^ T cells (T-ex^eff^, PD1^+^ act.) (cluster 2 in **e**). NKX6.1 marks β-cells. **e**, Pearson correlation coefficients of infiltration scores of the indicated stage-associated immune cell types from insulitic ROIs (≤20 µm to the islet edge). Numbers and brackets highlight immune cell motifs. Motifs contain myeloid subtypes (M). **f**, Mean immune cell numbers between insulitic infiltrates of < 13 year and ≥13 year donors (each dot shows mean per donor across insulitic islets). Violin lines are as in **b**. Tests: differential abundances were computed across age using edgeR (two-sided empirical Bayes quasi-likelihood *F*-tests). Significances are FDR adjusted. For **b**: controls: *N* = 15; sAAb+: *N* = 28; mAAb+: *N* = 10; Onset: *N* = 21. For **e** and **f**, mAAb+: *N* = 3; Onset: *N* = 14.

T1D severity varies across age, and the disease is known to be more precipitous in young patients^43^. To understand the potential basis of this effect, we sought to define potential age-associated immune cell motifs. We first compared the abundance of immune cell subtypes across disease stages between younger and older donors (Fig. 6a,b). This enabled us to identify immune cell subtypes that were (i) age associated, (ii) stage associated, (iii) age and stage associated or (iv) non-associated (Fig. 6a).

**Extended Data Fig. 10:**
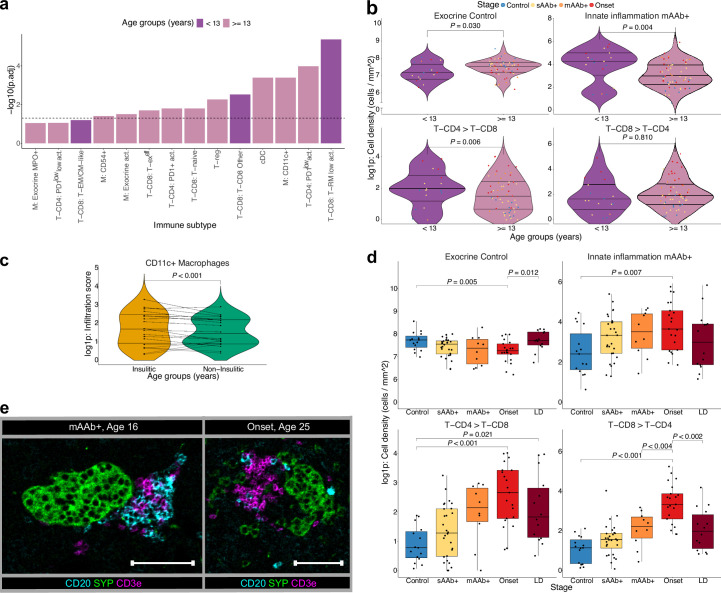
Spatial, progression, and age-associated immune cell motifs. **a,** Differential abundance model testing association of age with immune cell subtype abundances. The horizontal line indicates an adjusted *P*-value = 0.05. Input data is as in Fig. 6a. **b,** Violin plots of CN density between younger (<13 years) and older (≥13 years) donors (each dot shows the sample mean across ROIs; coloured by disease stage). y-axis break included for visualization. **c,** Violin plots of CD11c^+^ macrophages comparing densities between insulitic (i = 179) and non-insulitic (i = 552) ROIs from the same donors (N = 19); lines connect ROI pairs from the same donor. **d,** Boxplots of CN densities across diseased stages (each dot, donor means across ROIs). The center line indicates the median, box bounds represent the IQR, and the whiskers extend to 1.5x the IQR. y-axis break included for visualization. **e,** Representative micrographs from one mAAb+ (left) and one Onset donors (right) with substantial CD20^+^ B-cell (cyan) infiltration. Tests: (**a, b, d**): Differential abundance tested with edgeR (two-sided empirical Bayes quasi-likelihood *F*-test). (**c**) Differential infiltration score tested by linear-mixed effects model (two-sided Wald *t*-test with Satterthwaite approximation). (**b, d**) Control: N = 14; sAAb+: N = 28; mAAb+: N = 10; Onset: (**b**) N = 21 or (**d**) N = 18; LD: (**d**) N = 14.

We identified multiple immune cell types as age-associated (i, iii) with CD11c^+^ macrophages being strongly enriched in younger donors (Fig. 6a,b and Extended Data Fig. 10a). CD11c^+^ macrophages expressed the highest level of TMEM173 (STING) of all myeloid cells, suggesting that they are type 1 IFN producing cells^44^ (Fig. 5b) and often expressed HLA-DR (Figs. 5b and 6d), indicating increased capacity for antigen presentation. These cells were enriched in spatial neighbourhoods with high innate immune cell content (‘innate inflammation mAAb+’) (Fig. 5g), which were more abundant in younger donors, as expected (Extended Data Fig. 10b), as well as in T cell enriched CNs (‘T_CD4_ > T_CD8_’, ‘T_CD4_ < T_CD8_’) (Fig. 5g). In combination with their potential APC capacity and enrichment in insulitic infiltrates (Extended Data Fig. 10c), this also suggests interactions with T cells. Older donors had higher abundances of marginally activated tissue-resident memory T_CD8_ cells (T_RM_ low. act.) than did younger donors, at all disease stages (Fig. 6b). In comparison with other T_CD8_ subtypes, T_RM_ low act. cells were enriched in exocrine tissue CNs (‘exocrine control’) found more frequently in control donors and older donors (Extended Data Figs. 7c and 10b,d). This suggests a less diabetogenic profile of T_RM_ low act. cells compared with other T_CD8_ cell subtypes. Of note, while both T_RM_ low act. cells and CD11c^+^ macrophages were associated with both age and disease stages (iii), trends in their abundance were apparent also in controls (both *P* = 0.08), suggesting that these cell types contribute both to disease and to age-associated variations in the pancreas.

Next, focusing only on T_CD4_, T_CD8_ and myeloid cell subtypes that we identified as relevant for disease progression (ii, iii), we correlated infiltration scores in insulitic islets to identify infiltration motifs (that is, combinations of cell types infiltrating these islets) (Fig. 6c). We then compared abundance of the corresponding immune subtypes within these islets between younger (<13 years) and (≥13 years) donors.

We observed two major insulitic motifs (Fig. 6d,e). First, we observed that T_CD8_ naive and B cells formed a cluster (Fig. 6d,e, cluster 1), suggesting the presence of B cell-rich infiltrates or tertiary lymphoid structure (TLS)-like structures (Fig. 6d). B cells and T_CD8_ naive cells were at higher abundance in insulitic infiltrates of younger donors in our cohort (Fig. 6f), consistent with previous results^38,45^. This segregation across age was not complete, however, as examples of infiltrates with high B cell content in older donors could also be found (Extended Data Fig. 10e).

A second motif in insulitic islets comprised multiple myeloid subtypes that clustered with exhausted-like effector T_CD8_ (T-ex^eff^) cells, probably indicative of myeloid and T cell co-infiltration (Fig. 6d,e, cluster 2). This included not only M1/M2-like act. macrophages, as described above (Fig. 5i) but also CD11c^+^ macrophages. Comparison of insulitic infiltrates between younger and older donors showed no strong differences for T-ex^eff^ cells and M1/M2-like act. macrophages (*P* > 0.47), suggesting that this immune motif is present across age (Fig. 6f). This also applied to other key immune cell types (PD1^+^ act. cells and cDCs) that co-infiltrated insulitic islets (Fig. 6e,f, cluster 3). CD11c^+^ macrophage abundance, however, trended higher in insulitic islets of younger donors (*P* = 0.05), again suggesting additional age-associated effects.

In summary, analysis of immune cell type abundance across disease stages and age showed that CD11c^+^ macrophages and marginally activated T_RM_ were the main immune cells that varied by age, with potential implications for homeostasis and T1D disease. Insulitic infiltrates comprising M1-polarized macrophages and T-ex^eff^ cell motifs were observed across all ages, whereas B cell-rich/TLS-like structures were enriched in younger donors, which might be associated with differential disease severity between younger and older donors.

## Discussion

T1D progression involves a complex interplay among endocrine, adaptive immune and innate immune cells^3^. We report here a first of-its-kind cellular-resolution multiplexed imaging study of pancreatic samples from 88 donors across all stages of T1D, including sAAb+ and mAAb+, matched by age, sex and BMI. Our analysis thus enabled interrogation of the human β-cell state and the pancreatic islet–immune interface associated with T1D progression at unprecedented scale.

Our analysis showed that human pancreatic β-cells are dysregulated before clinical disease onset. Most strikingly, we detected lower levels of the IAPP hormone in mAAb+ donors, a stage that precedes loss of co-secreted insulin^34^, and therefore observed IAPP-INS+ islets. Undetectable IAPP might be explained not only by loss of IAPP protein but also by structural changes, as IAPP is aggregation prone. Furthermore, it might be indicative of a more general stress response in β-cells^46^. In support of this notion, β-cells in diabetic Onset donors showed higher levels of MHC-I and lower levels of 24 out of 28 β-cell lineage and functional markers in comparison to control donors. Our data offer several hypotheses of how these β-cell phenotypic changes might link to disease progression. Higher levels of MHC-I expression are probably detrimental to β-cell survival^7^, and reduced expression levels of functional markers (for example, TXNIP) could establish a heightened susceptibility of β-cells to stress pathways, such as chronic ER stress^46^. On the other hand, lower levels of lineage markers, and thus a more de-differentiated β-cell state, might not only confer a survival bias but also reduce β-cell function^47^.

Previous work has reported signs of heightened ER stress in T1D samples^20–22^, but we did not observe this for the ER stress markers WFS-1, IRE1α-P and XBP1 in both cross-sectional and intra-donor comparisons. Further, a comparison of α-cells between ICIs (islets where β-cells were present) and IDIs (islets where β-cells were depleted) within the same donors also showed similar or even lower levels of IRE1α-P in ICIs, despite these probably being more inflamed. Based on the markers we have analysed (XBP1, WFS-1 and IRE1α-P), we therefore do not find evidence for increased ER stress in T1D in this cohort. We note that recent studies of human T1D using different tissue biobanks and different technologies also support these findings^48–50^. We recognize that our analysed markers have limitations: we were unable to include BiP and CHOP in our study because we could not find high-quality antibodies targeting these proteins (Supplementary Table 5), a problem that been previously discussed^21^. Furthermore, we measured total XBP1 rather than only the sXBP1 isoform, which is a more accurate ER stress correlate. However, reanalysis of a published scRNA sequencing dataset supported our findings by showing no evidence for upregulation of BiP and CHOP and other markers in T1D at the transcript level and by showing absent heightened activity of XBP1 and CHOP or indeed of any measured ER-stress-associated TFs in T1D donors. Nevertheless, our conclusions regarding ER stress necessarily apply only to markers we measured in this study and not to ER stress in general.

Although most β-cells in Onset donors displayed higher MHC-I levels and overall lower protein levels than in controls, β-cells within insulitic islets specifically expressed MHC-II and showed higher levels of IFN-responsive markers. This is probably caused by a shift to an IFN-γ driven cytokine environment^7^ that we observed during the infiltration of exhausted-like T cells, which in turn may precipitate infiltration of further non-exhausted T cells, for example, via ICAM-1 upregulation. The insulitic-specific β-cell state might facilitate direct antigen presentation between β-cells and T_CD4_ cells but could also reflect β-cell protective effects^51^. Although effect sizes were often largest in β-cells, we observed similar marker changes with disease stage in other endocrine and islet-proximal exocrine cells both for MHC-I, with significantly higher levels in α-cells already in mAAb+ donors, as well as for MHC-II. This suggests general islet-wide and islet-proximal cytokine effects both before and after disease onset and raises the question why β-cells, but not other endocrine or islet-proximal cells, are killed^52^. Here, comparisons to α-cells but also to δ-cells and islet-proximal exocrine cells will be informative.

In parallel, we analysed immune cell abundances and infiltration across T1D disease stages. The earliest detected perturbations in the islet–immune interface of the human pancreas were HLA-ABC expression in β-cells, as well as infiltration of M1/M2-like act. macrophages and T_CD8_ T-ex^eff^ cells, and islet-proximal infiltration of T_CD4_ PD1^+^ act. cells. These immune cell subtypes trended higher already in sAAb+ donors and, with T_reg_, were the only subtypes enriched at this early disease stage, indicating their importance for early disease progression and suggesting that they may interact. Consistent with this, in mAAb+ and Onset donors, we observed several indicators of interactions between myeloid cells (mainly M1/M2-like act. macrophages and cDCs) and exhausted-like T_CD4_ and T_CD8_ cells (PD1^+^ act. cells and T-ex^eff^ cells). Notable interactions were observed both at the islet edge in mAAb+ donors and during overt islet infiltration, key stages of disease progression. We also detected co-stimulatory and APC phenotypes, such as M1-polarized macrophages and mature cDCs, suggesting antigen presentation and co-stimulation to T cells^15^. These signals might be required to deliver survival signals to T cells and/or subsequent effector programme acquisition for precursor exhausted T cells (T_PEX_), which have been attributed a key role in T1D and might be present within the exhausted-like T cell populations we have here described^15,53^. Nevertheless, while these interactions imply diabetogenic effects, the high levels of TIM-3 on cDCs suggest an immuno-regulatory phenotype that might slow T1D progression^42^, and the abundant interactions between macrophages with exhausted-like T cells also suggest a role in T cell exhaustion, as observed in tumour immunity^54^. Based on these data, we propose that the role of macrophages and DCs in the human pancreas to T1D extends beyond cytokine secretion and that instead, interactions of macrophages and DCs with exhausted-like T cells govern not only key diabetogenic but also protective effects in T1D progression. Furthermore, myeloid cells and exhausted-like T cells may be targeted by immune checkpoint inhibitors in cancer therapy, which may explain the reported adverse induction of T1D^55^. This would apply not only to currently approved PD1 treatment targeting exhausted-like T cells but also to anti-TIM-3 treatment potentially targeting both islet-infiltrating TIM-3^+^ exhausted-like T cells and the detected TIM-3^high^ cDCs^42,56^.

Children present with more aggressive disease than adolescents and young adults^43^; thus, we analysed our data for age-dependent effects. Insulitic infiltrates containing exhausted-like T cells and pro-inflammatory macrophages were present across age. This further underlines their importance to disease and highlights the clinical potential to modulate this axis in patients with T1D across age to enforce exhaustion in T cells. Further, we observed that immune cells of TLS-like structures (that is, B cells and naive T_CD8_ cells) were enriched in younger donors (<13 years) in comparison with older donors (≥13 years), akin to a previously proposed stratification (CD20^high^: <7 years, CD20^low^: ≥13 years, mixed: 7–12 years)^38^. Nevertheless, we also detected (albeit rarely) B cell-high areas in some older donors, which could refine this stratification. Although a limitation of our study is that it included fewer younger than older donors, our data are supported by previous results in which islet-associated TLS-like structures were also observed in donors older than 12 years of age^45^.

Across disease stages, including in non-diabetic controls, we observed CD11c^+^ macrophages and marginally activated T_CD8_ as the main cell types that differed in abundance between younger and older donors. These differences thus probably reflect the evolution of the pancreatic immune niche composition across age, with potential implications for T1D disease. Due to high STING protein levels on CD11c^+^ macrophages, we hypothesize these cells to exhibit an IFN-mediated anti-viral innate immune programme in young donors, as was observed in the upper airways^57^. This is highly relevant (1) as viral infections modulate risk for auto-immunity onset^58^ and (2) SNPs in innate dsRNA pattern-recognition receptors such as *IFIH1* are known risk-factors for progression to T1D disease^59,60^. Further studies using healthy as well as diseased pancreata, in the context of T1D or viral infections, should establish function of these cells and verify T1D-specific effects, for example, by an aberrant activation of these cells in younger donors.

Our study has limitations. First, IMC is limited by data acquisition speed, thus making unfeasible the imaging of whole-slide fields-of-view at cellular resolution and high multiplex (*N* = 79). We therefore focused on imaging many (*N* = 75) small ROIs per tissue section, choosing these ROIs to include islets and proximal exocrine tissue, to strike a balance between data acquisition time and informative data content. This is at the expense of unbiased detection of islets of all sizes as well as detection of single islet cells dispersed in the exocrine tissue. Specifically, our ROI and segmentation strategies resulted in an under-sampling of smaller islets (~40 µm diameter)^61^ (Supplementary Fig. 1a) and did not define small endocrine masses <8 µm diameter as islets. Nevertheless, this effect was small, as was confirmed by capturing expected islet-size dependent effects across disease stages that align with recent studies^62,63^ (Supplementary Fig. 1b–d).

A second limitation is that we did not derive antigen specificity of T cells. Third, studying longitudinal progression of a disease by cross-sectional comparisons of donor tissue sections is inherently limited, as these samples are post-mortem snapshots of disease, potentially influenced by end-of-life effects and organ handling^33^. Nevertheless, it is currently the only feasible option for large-scale studies on human pancreatic tissue. Fourth, samples of each disease stage in our cohort were matched for most measured variables, except for organ transit time and cause of death. We ensured that these had no strong effect on the main cell types and processes (that is, immune cell abundance and β-cell markers). Instead, we identified ‘ICU time’ to be (weakly) associated with immune cell abundance and have thus regressed out this effect throughout the analyses. We note as well, that the smaller mAAb+ group (*N* = 10) included only a single patient <13 years, as such organ donors are (fortunately) rare. Finally, due to clinical reality, type 1 diabetics, but not controls, had a high genetic risk profile (that is the GRS2 score) and were treated with exogenous insulin. These features should be considered in any future analysis of this dataset, as treatment or higher genetic risk may introduce changes to the islet–immune interface.

We envision that in combination with the present study, future multimodal studies of human pancreata from subjects with diverse genetic backgrounds could facilitate creation of T1D disease atlases that can be used to associate disease features with clinical covariates and intervention outcomes and thereby help developing personalized strategies to treat T1D.

## Methods

### Donor details

Pancreatic samples were obtained from nPOD in compliance with all relevant ethical regulations. Procedures were approved by the United Network for Organ Sharing (UNOS) according to federal guidelines, and informed consent was obtained from each donor’s legal representative. Written informed consent to publish medical information potentially identifying individuals was obtained from all their representatives. All tissue samples, as well as corresponding clinical information, were collected after approval by the University of Florida Institutional Review Board (IRB201600029). Gender information was not collected. Sex information is as reported in the donor sheet; filled out by the donor’s legal representative. Donors were not compensated financially. Donors included subjects with sAAb+, mAAb+, recent-onset (Onset) T1D (≤2 years), long-standing (LD) T1D (≥3 years) and autoantibody-negative controls without diabetes. Cases were matched by BMI, sex and age across batches and disease stages (Extended Data Fig. 1). Full donor information is listed in Supplementary Table 1. Seven donors with Pancreatitis were additionally measured. These data are available for reproducibility purposes but they were excluded from downstream analysis.

### Method details

#### Antibody testing and validation

Antibodies were initially tested with immunofluorescence. Antibodies targeting immune markers were tested on lymphoid tissues (spleen, tonsil and lymph node) and antibodies targeting pancreas markers were tested on pancreatic tissue. Markers displaying expected expression patterns were conjugated to heavy metals and tested by IMC.

#### Pilot study

Formalin fixed paraffin embedded (FFPE) pancreatic tissue sections from 16 organ donors were stained (controls, *N* = 4; sAAb+, *N* = 4; mAAb+, *N* = 4; Onset, *N* = 4) with a total of 130 antibodies in five separate IMC panels (Supplementary Tables 4–8). Antibodies were selected for the inclusion in the final panels based on expected staining patterns, high signal-to-noise ratio and non-redundancy.

#### Antibody conjugation and titration

Metals were conjugated to all antibodies using the MaxPar X8 Multimetal Labeling Kit (Fluidigm) according to the manufacturer’s instructions. FFPE pancreatic tissue sections were stained with serial dilutions of relative concentrations of 1,500, 1,000, 500, 250 and 125. One section was used per dilution. Pancreatic sections were imaged by IMC, and raw IMC data were processed by steinbock^64^ (v. 0.10.2). The signal-to-noise ratio was calculated by defining positive and negative cells. The dilution with the highest signal-to-noise ratio was selected for each antibody.

#### Tissue staining

Per organ donor, two consecutive 4-µm pancreatic tissue sections were stained, one with the islet panel and one with the immune panel (Supplementary Tables 2 and 3).

FFPE tissue sections were deparaffinized and rehydrated using a graded alcohol series. Antigen-retrieval was performed in a decloaking chamber for 30 min at 95 °C in HIER buffer (pH 9.2), and tissues were blocked with 10% normal horse serum. Sections were first incubated for 1 h at 4 °C with unconjugated primary antibodies (immune panel: mouse anti-CD3e, rabbit anti-PD1; islet panel: hyaluronic acid binding protein conjugated with biotin, mouse anti-CD45RA, mouse anti-CD45RO and rabbit anti-PD-L1). Next, sections were stained for 1 h at room temperature with conjugated secondary antibodies (immune panel: anti-mouse IgG-^152^Sm, anti-mouse IgG-Alexa Fluor 555, anti-rabbit IgG-^166^Er; islet panel: anti-biotin IgG-^115^In, anti-rabbit IgG-^160^Gd, anti-mouse IgG-Alexa Fluor 555) and counterstained with Hoechst. Pancreatic tissue sections were then incubated with metal-conjugated primary antibodies at room temperature for 3.5 h (islet panel: 13 antibodies; immune panel: 17 antibodies) (Supplementary Tables 2 and 3) and incubated overnight at 4 °C with the remaining metal-conjugated primary antibodies and mouse anti-CD99 allophycocyanin.

At this stage, immunofluorescence (IF) images were acquired, and the slides were counterstained with iridium DNA intercalator for 5 min at room temperature before air drying using pressurized air.

#### ROI selection

Brightfield and IF images of the pancreatic sections were acquired on a slide scanner (Zeiss Axio Scan.Z1) at 2.5× and 10× magnification, respectively. The IF images of the immune panel (CD3e, CD99 and DAPI) were automatically registered to the immunofluorescent images of the islet panel (CD45, CD99 and DAPI) in FiJi^65^ (v. 1.5.3p) using the virtual stack slices registration plugin (v. 3.0.7).

ROIs were selected based on the islet panel immunofluorescent images, as follows. First, islets were segmented by training a pixel classifier in ilastik^66^ (v. 1.3.3post3) and labelling pixels as either islet or exocrine and background. Pixel probability maps were then exported to CellProfiler^67^ (v. 4.2.1) to segment islets. Islet masks were imported into FiJi and loaded as a set of ROIs using rectangular bounding boxes. Bounding boxes of the islet panel and immune panel were expanded by 50 µm and 80 µm, respectively. Equal tissue areas across panels for IMC ablation were selected by first recording ROI coordinates on the islet panel and then transforming the ROI coordinates to the immune panel. ROIs focused on imaging islets, their proximal exocrine tissue and their infiltration by immune cells. Imaged islets were sampled evenly across tissue position and immune infiltration but showed under-sampling of structures of 40–50-µm diameter (Supplementary Fig. 1a).

#### IMC data acquisition

The consecutive sections, respectively stained by the islet and immune panels, were acquired on two separate Helios time-of-flight mass cytometers (CyTOF) coupled to Hyperion Imaging Systems (Fluidigm). Laser ablation was set at 200 Hz and resolution was 1 µm. Before acquisition, optical images of slides were acquired using the Hyperion software, and IF ROI coordinates were translated to IMC ROI coordinates by manually selecting landmarks and performing image registration.

Data were acquired in four batches in a randomized order (Extended Data Fig. 1a), with a variance minimization approach to minimize inter-batch differences in patient covariates^68^. Performance stability was ensured by daily calibrating the mass cytometers with a tuning slide spiked with five metal elements (Fluidigm). For each section, 75 ROIs were acquired per panel. This yielded, after quality control, 14,050 47-plex image stacks from 88 pancreas sections.

### Quantification and statistical analysis

A multi-step preprocessing pipeline was implemented, which included reading of raw IMC files, islet segmentation, cell segmentation, image registration and feature extraction (Extended Data Fig. 2a).

#### IMC data processing

Raw IMC.txt and.mcd files were converted to.tiff images using the preprocess functionality of steinbock (v. 0.15) with a hot pixel threshold of 50.

#### Islet segmentation

IMC data from the pilot study were used to generate training islet masks via ilastik and CellProfiler using only informative markers during pixel classification (Supplementary Table 9). Resulting islet binary masks were used to train a deep convolutional neural network using PyTorch (v. 1.13.1) (images: 67% training, 33% test, donor-stratified). For inference of islet segmentation masks from the cohort, expression of informative channels (Supplementary Table 9) was min–max normalized and aggregated. Images were resized (160 × 160 pixels), and islets smaller than 50 pixels (50 µm^2^) with an eccentricity above 0.95 were not considered. Binary holes in the islets were filled.

Images and masks were visually inspected, and images with (1) bad image quality, (2) mismatched acquired regions on consecutive sections, (3) disrupted acquisition due to software crash or experimental handling or (4) non-segmented islets were not included in downstream analyses. Over 11,000 islets were segmented and labelled (Extended Data Fig. 2b,c).

#### Cell segmentation

Whole-cell segmentation masks were generated using Mesmer^69^ as implemented in steinbock (v. 0.16). Informative nuclear markers and membrane or cytoplasmic markers (Supplementary Tables 2 and 3) were min–max normalized, aggregated and used as input for the Mesmer model. Approximately 6.2 million and 10.5 million cells were segmented in the islet and immune panel, respectively (Extended Data Fig. 2b,c).

#### Object measurements

The following features were extracted using functionalities from steinbock^64^: (1) average marker intensities, (2) region properties such as area, position and eccentricity, (3) the distance of cells to the islet edge and (4) cellular neighbours. 1 and 2 were extracted from both islets and cells.

#### Spillover compensation

Channel crosstalk was removed on the single-cell level by performing spillover compensation^70^. For each acquired batch, a slide was spotted with the same metals used for antibody conjugation and was measured with IMC. The generated spillover matrices were used to correct signal spillover with the Catalyst package^70^ (v. 1.26.1) (Extended Data Fig. 2d).

#### Data transformation and normalization

Single-cell expression counts were normalized by (1) arcsine transformation (co-factor of 1), (2) batch correction of arcsine-transformed counts using the fastMNN functionality from the batchelor package^71^ (v. 1.18.1) or (3) minimum–maximum normalization and 99% percentile clipping. Unless stated otherwise, arcsine-transformed counts (that is, approach 1) were used.

#### Cell type annotation

To identify single-cell phenotypes, cells were clustered separately per antibody panel using a multi-step approach. If not indicated otherwise, within each step four separate annotations were performed, by combining two unsupervised clustering approaches with two distinct data normalizations.

Unsupervised clustering was carried out: by using the PhenoGraph algorithm, as implemented in the Rphenoannoy package^72^ (v. 0.1.0), and by constructing a shared-nearest neighbours graph and detecting communities using the Leiden algorithm as implemented in the igraph package (v. 2.0.3)^73^. Scaled counts and fastMNN-embedded counts were used as input for the PhenoGraph and the Leiden algorithm.

Single cells were manually annotated to a cell type if the same cell type was predicted in three out of four annotations. Otherwise, cells were annotated as ‘ambiguous’. Expected marker expression of annotated cell clusters were visualized with cytomapper^74^ (v. 1.2.0), cytoviewer^75^ (v. 1.14.0) and napari (v. 0.4.17).

##### Islet panel annotation

Islet panel annotation was performed in three steps. First, cells within the islet mask were annotated as α, β, δ, ε, γ, and ‘other’ cells. Second, endocrine cells outside of the islet mask were predicted and annotated using a random forest classifier as implemented in ranger^76^ (v. 0.16.0), using the previously annotated endocrine cells as labelled training data. Finally, non-endocrine cells were annotated as ductal, acinar, endothelial, mesenchymal, T cells, macrophages and ‘other’. Used markers are shown in Supplementary Table 10.

##### Immune panel annotation

Immune panel annotation was performed in five steps. First, cells were separated into either immune or non-immune cells. Second, all non-immune cells were annotated as α, β, δ, ‘islet-other’, acinar, ductal, nerve, endothelial, smooth muscle and ‘other’. Third, immune cells were separated into major immune categories (that is, lymphocytes, neutrophils and myeloid cells). Fourth, lymphocytes were classified into B, NK, T-CD4, T-CD8, T-DN, and CD303^+^/VIM^+^ cells. Finally, T_CD8_, T_CD4_, myeloid cells, neutrophils, and NK cells were sub-clustered. Used markers for each step are shown in Supplementary Table 11.

As total protein levels (including FoxP3) are strongly associated with activation in T cells^77^, we observed strong correlation of FoxP3 to CD3e protein levels (T_CD8_ cells, *r* = 0.78; T_CD4_ cells, *r* = 0.67). This prevented reliable annotation of T_reg_ (FoxP3^high^) during clustering. To distinguish FoxP3^high^CD3e^mid^ cells (T_reg_) from FoxP3^high^CD3e^high^ (activated conventional T cells), we used generative additive models to model the FoxP3 upregulation in activated T cells. We assigned T_reg_ based on high FoxP3 expression (1> arcsinh-scaled counts) and large FoxP3 expression model residuals (greater than the 99th quantile of T_CD8_ FoxP3 model residuals).

#### Statistics and reproducibility

Statistical analyses were performed using R (v. 4.5.1) and tests were adjusted for multiple comparisons by false-discovery rate (FDR) correction^78^. As 75 IMC ROIs were acquired per organ donor, ROI-level aggregation and linear mixed-effect models (LMM) were applied to test for differential expression and differential infiltration scores between disease stages. LMMs with random intercepts were fitted using the lmerTest package^79^ (v. 3.1.3) with the case ID of each donor as a random effect. Differential abundance tests were carried out using edgeR^80^ (v. 4.0.16). Comparisons between disease stages were computed using the emmeans package. We did not compute comparisons that were non-consecutive and non-baseline (for example sAAb+ versus Onset).

Recent work has suggested the existence of age-associated endotypes^23^. Our cohort included donors aged <7 years (*N* = 11), 7–12 years (*N* = 9) and ≥13 years (*N* = 68). We merged donors <13 years into a single category (*N* = 20). For β-cells, we fit two separate LMMs to estimate the influence of age.

The first model was just applied to Onset T1D organ donors

$$Y\sim {\mathrm{disease}}\_{\mathrm{duration}}+{\mathrm{age}}\_{\mathrm{group}}+(1|{\mathrm{case}}\_{\mathrm{id}}).$$

The second model was applied to control, sAAb+, mAAb+ and Onset T1D donors

$$Y\sim {\mathrm{disease}}\_{\mathrm{stage}}+{\mathrm{age}}\_{\mathrm{group}}+(1|{\mathrm{case}}\_{\mathrm{id}}).$$

Y denotes normalized marker expression. Disease duration is the time since onset, and age_group denotes the two age groups defined above (<13 years; ≥13 years). We excluded LD donors from this analysis, as there was a considerable time lag between age of onset and age of donation (up to 21 years), probably skewing age-associated effects. To study associations to GAD autoantibody titres, we adjusted for age_group and cause of death, to adjust for potential titre-biasing effects of blood transfusions in patients with trauma.

For immune cells, we observed strong age-associated effects, as well as association of the covariate ICU time with densities of some immune cell types (Supplementary Fig. 6b). We thus introduced age as covariate and ICU time as a blocking variable in cross-sectional comparisons. This captures variance explained by ICU time and not by disease stage. Hence, linear mixed-effects models to for example test infiltration scores between disease stages were

$$Y\sim {\mathrm{disease}}\_{\mathrm{stage}}+{\mathrm{age}}\_{\mathrm{group}}+{\mathrm{ICU}}\_{\mathrm{time}}+(1|{\mathrm{case}}\_{\mathrm{id}}),$$

where *Y* denotes infiltration score, immune cell type density or expression.

IMC is a destructive technique, and tissue sections were thus acquired once. Shown micrographs are representative of repetitive tissue structures, and the given biological trends are always quantified in other subpanels. Data distribution was assumed to be normal, but this was not formally tested. Data distributions per donor are shown. Data collection and analysis were not performed blind to the conditions of the experiments. Due to the severe scarcity of measured human AAb+ samples, we measured all available AAb+ samples. Thus, no statistical methods were used to predetermine sample sizes.

#### Pseudotime analysis

Pseudotime analysis of β-cell expression profiles was performed using slingshot^32^ (v. 2.10.0). We used default parameters to identify the global lineage structure and fit smooth branching curves to infer the pseudotime variables for each β-cell. Notably, we did not provide start and leaf nodes or cluster information and thus, slingshot inferred the global trajectory in an unbiased manner. As input, we used diffusion map embedded single-cell β-cell expression profiles, which were computed using destiny^81^ (v. 3.16.0). We considered the expression of 28 β-cell lineage and functional markers for computation of the diffusion map and pseudotime (Supplementary Table 10). The association of β-cell markers to pseudotime was computed using tradeSeq^82^ (v. 1.16.0). We computed cross-correlations and Granger causality along pseudotime using the stats and lmtest (v. 0.9.40) package, respectively.

#### Infiltration score

The infiltration score was assigned to every cell by assigning a value of ‘1’ to islet-infiltrating cells and a distant-dependent value to cells outside the islet. By weighting for distance, this score rewards abundance increases in the peri-islet space and penalizes abundance increases in tissue areas distal to the islet. The infiltration score is defined as

$$g({d}_{i,\,j})=\left\{\begin{array}{cc}\frac{1}{\sqrt{d}}, & \mathrm{if}\,d\ge 1\\ 1, & \mathrm{if}\,d < 1\end{array}\right.$$

$$\mathrm{Infiltration}\,{\mathrm{score}}_{\mathrm{case\_id}}=\frac{1}{M}\mathop{\sum }\limits_{j=1}^{M}\mathop{\sum }\limits_{i=1}^{N}g\left({d}_{i,\,j}\right)\times \left(1+\frac{{\mathrm{diameter}}_{\mathrm{stage}}}{{\mathrm{diameter}}_{j}}\right).$$

Here, *d* is the distance of the immune cell *i* from the edge of the islet *j* in micrometres, diameter indicates the islet diameter, with constant diameter_stage_ adjusting for any stage effects. The infiltration score per donor is averaged across all *M* islets.

#### Spatial graph

Two spatial neighbourhood graphs were built for each image by using imcRtools^64^ (v. 1.8.0). One contained the five nearest neighbours within a 15-µm radius, and one graph considered the ten nearest neighbours within a 25-µm radius. Cells 10 µm from the image edge were removed to exclude edge effects. imcRtools was used both to aggregate the cellular composition and expression of neighbouring cells.

#### CN analysis

We applied CN analysis to the immune panel data to identify spatial tissue areas of defined cell type composition^40^. We used *k*-means clustering to aggregate the cellular composition of the ten nearest neighbours of each cell. Given that highly relevant biological processes such as T cell infiltration are rare and probably change across disease progression, we hypothesized that a high number of CNs would be required to capture the biological complexity of T1D progression. Therefore, we tested multiple settings for *k*. We estimated cluster stability with the Silhouette score and tested for biological relevance by calculating the association of each CN fraction to disease progression and identified *k* = 70 as the ‘optimal’ setting. CNs were manually annotated by their enriched cell types and their localization relative to the islet. We merged CNs if we observed homogeneous cell type distributions between clusters, and similar fractions across disease stages. We explicitly did not merge T cell-enriched CNs, as we assumed their relevance for disease.

#### Lead contact

Requests for further information and resources should be directed to and will be fulfilled by the lead contact, B.B. (bernd.bodenmiller@uzh.ch).

#### Materials availability

Additional tissue samples from donors evaluated as a part of this study can be requested from nPOD for use in projects approved by the nPOD Tissue Prioritization Committee, as outlined on the nPOD website (https://npod.org/). This study did not generate new unique reagents.

### Reporting summary

Further information on research design is available in the Nature Portfolio Reporting Summary linked to this article.

## Supplementary information

Supplementary Information Supplementary Figs. 1–7.

Reporting Summary

Supplementary Tables Supplementary Tables 1–11 describing Organ Donors (Supplementary Table 1), antibody panels (Supplementary Tables 2–8) and informative markers used in preprocessing and downstream analysis (Supplementary Tables 9–11).

## Data Availability

All source data including the raw IMC.mcd files, convolutional neural network islet segmentation model, cell and islet masks, .tiff image stacks as well as annotated single-cell objects will be available via Zenodo at 10.5281/zenodo.14968076 (ref. ^27^). Annotated.tiff image stacks (for example ‘insulitis’) will be uploaded via Pancreatlas at https://pancreatlas.org/ (ref. ^28^). SCEs, .tiff image stacks and masks for fast import into R will be available via the imcdatasets (https://bodenmillergroup.github.io/imcdatasets/index.html) package at release.

## Code availability

Code to perform data processing and downstream analysis is available via GitHub at https://github.com/BodenmillerGroup/T1D_Publication_Steenbuck-Damond. This includes a Snakemake pipeline covering preprocessing and quality control.
