# Supplementary material for: Imaging mass cytometry reveals functional and immunological changes during type 1 diabetes progression in human pancreata
Source: Nat Metab. 2026 Jul 2;8(7):1603–20. doi: 10.1038/s42255-026-01559-z (PMC13400304; doi:10.1038/s42255-026-01559-z)
Supplement: Supplementary file 1 — Supplementary Figs. 1–7. [file 42255_2026_1559_MOESM1_ESM.pdf]

nature metabolism

Supplementary information https://doi.org/10.1038/s42255-026-01559-z

Imaging mass cytometry reveals functional and immunological changes during type 1 diabetes progression in human pancreata

In the format provided by the authors and unedited

Supplementary Figures:

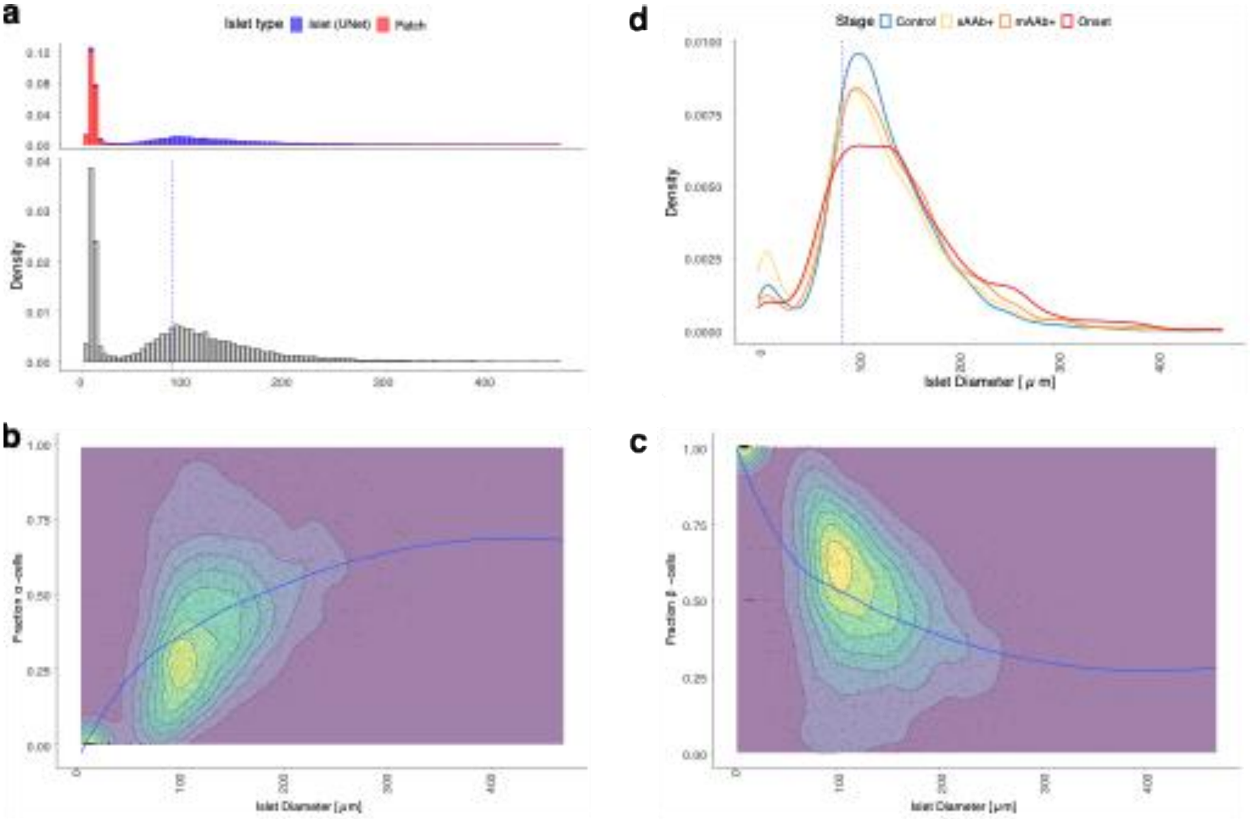

Supplementary Figure 1: *Stage and composition-dependent effects of islet size*. **a:** Lower panel: Joint islet size distribution of β-cell masses as measured by both our islet-defining UNet (≥ 50 μm², ~ 8 μm diameter, < 0.95 eccentricity) and the patch-detection algorithm, which detects all cells annotated as islet cell types outside of UNet-classified islets. Islet size is shown as islet diameter for comparison to reference values from 3D studies. The blue vertical line is the average islet diameter (88 μm) in the ROIs analyzed in this study. Islets were approximated as circles to compute the diameter. Upper panel: islet size distributions of islets and patches. **b, c:** The **(b)** α-cell and **(c)** β-cell fractions for β-cell containing patches and islets (ICIs). Fitted lines are LOWESS curves with 95% CI. Each dot is the diameter of one single islet or patch. α-cells are enriched in larger ICIs, whereas β-cells are enriched in smaller ICIs, as expected. **d:** Diameter distribution of β-cell containing islets and patches (ICIs) colored by disease stage. Smaller ICIs are preferentially lost in comparison to Control donors, as expected.

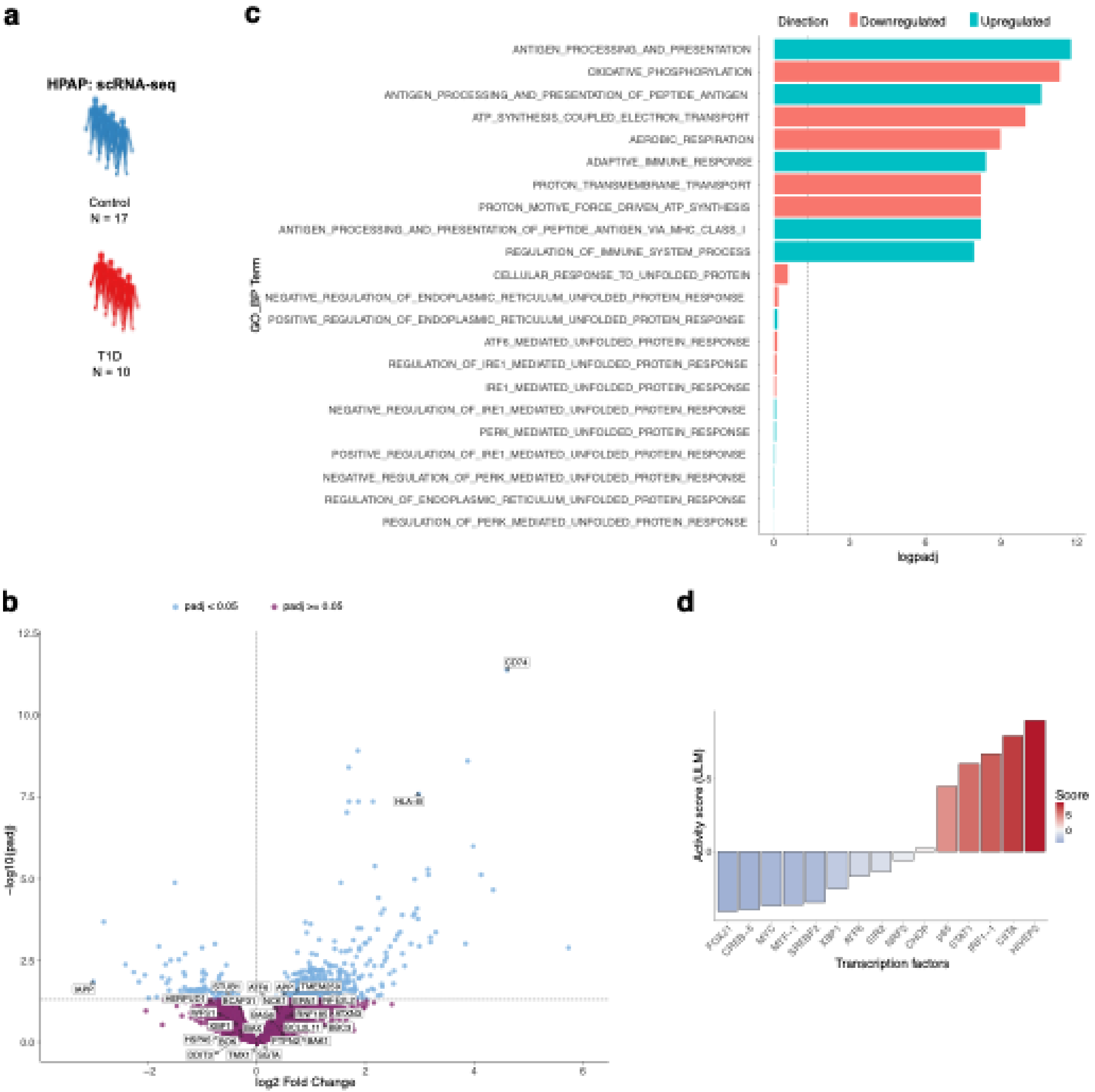

Supplementary Figure 2: *Reanalysis of a publicly available scRNA-seq dataset.* **a:** Overview of the tested Human Pancreas Analysis Program (HPAP) study population. **B:** Differential expression analysis between β-cells of controls (N=17) and T1D (N=10) HPAP donors. Tests were performed as in the original publication**.** Each dot is the differential expression and respective adjusted *P*-value per gene. Key markers (*IAPP, CD74, HLA-B*) and ER stress markers are indicated. **c:** Gene set enrichment analysis using GO terms. The top five upregulated and top five downregulated GO terms (ordered by adjusted *P*-value) are shown, as well as all available unfolded protein response (UPR) GO terms. The vertical line indicates the significance threshold of an adjusted *P*-value of 0.05. Tests were two-sided. **d:** Transcription factor activity analysis using CollecTRI regulons and a univariate linear model (ULM) to compute transcription factor activity scores**.** The transcription factors with the top five highest and lowest activity scores are shown, as well as all ER stress transcription factors (XBP1, ATF6, SIR2, NRF2, CHOP) included in the CollecTRI regulons. Transcript factor activity scores above 0 indicate higher activity in T1D donors.

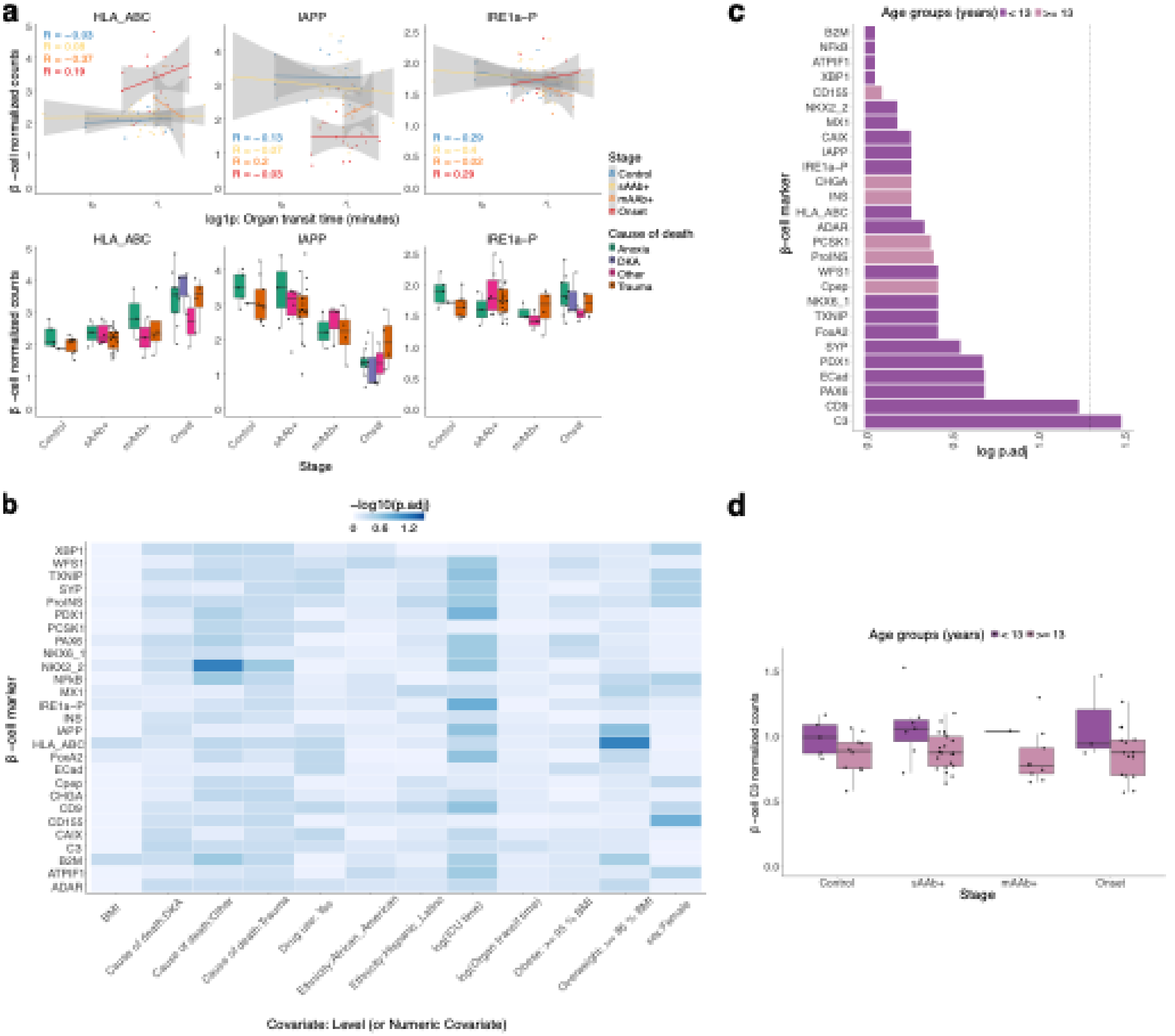

Supplementary Figure 3: *No covariate is associated with β-cell marker levels.* **a:** The asinh-normalized counts of key β-cell markers (HLA-ABC, IAPP, IRE1α-P) across disease stages, visualized against organ transit time and cause of death. Each dot indicates data from one donor with expression averaged across ROIs. The center line indicates the median, the box bounds represent the inter quartile range (IQR) from the 25th to the 75th percentile, and the whiskers extend to the minimum and maximum values within 1.5 times the IQR. Indicated *R* values are Spearman correlation coefficients. Error bands are the 95 % confidence interval (CI). **b:** Heatmap of the associations of covariates to all β-cell marker levels. Categorical variables are separated by colons from their respective levels, e.g., ('Drug use:Yes'). **c:** Significance of expression of indicated markers in a linear mixed-effects model comparing β-cell counts from donors < 13 years and ≥ 13 years across disease stages. Control, sAAb+, mAAb+, and Onset donors were included. Color indicates higher expression levels in either the < 13 year age group or ≥ 13 year age group. **d:** The asinh-normalized β-cell complement component 3 (C3) counts by age groups across disease stages. Each dot indicates data from one donor with expression averaged across ROIs. Center line, box bounds, and whiskers are as in panel **(a)**. Tests: The following linear mixed-effects model were fit: *Expression ~ disease_stage + age_group + covariate + (1|case_id).* FDR-adjusted significances were computed using two-sided Wald t-test with Satterthwaite approximation. *P*-values were adjusted separately by covariate to avoid *P*-value deflation in comparison to tests against one specific covariate. Control: N=15; sAAb+: N=28; mAAb+: N=10; Onset: N=21.

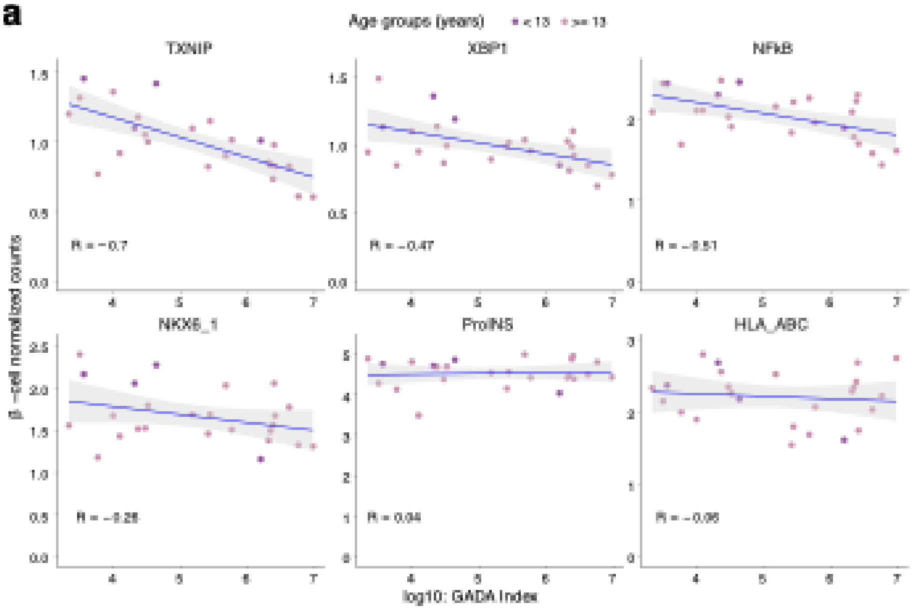

Supplementary Figure 4: *Association of GAD titers with β-cell marker levels.* **A:** The asinh-normalized counts of key β-cell markers correlated against GAD autoantibody (GADA) titers for GADA+ sAAb+ donors (N = 25). GADA titers were tested against all β-cell markers and the markers with the strongest association (TXNIP, XBP1, NF-κB) are shown; also shown are NKX6-1, ProINS, and HLA-ABC as examples of key markers without any association. The indicated *R* value is the Spearman correlation coefficient. Each dot indicates data from one donor with expression averaged across ROIs. Error bands are the 95% CI.

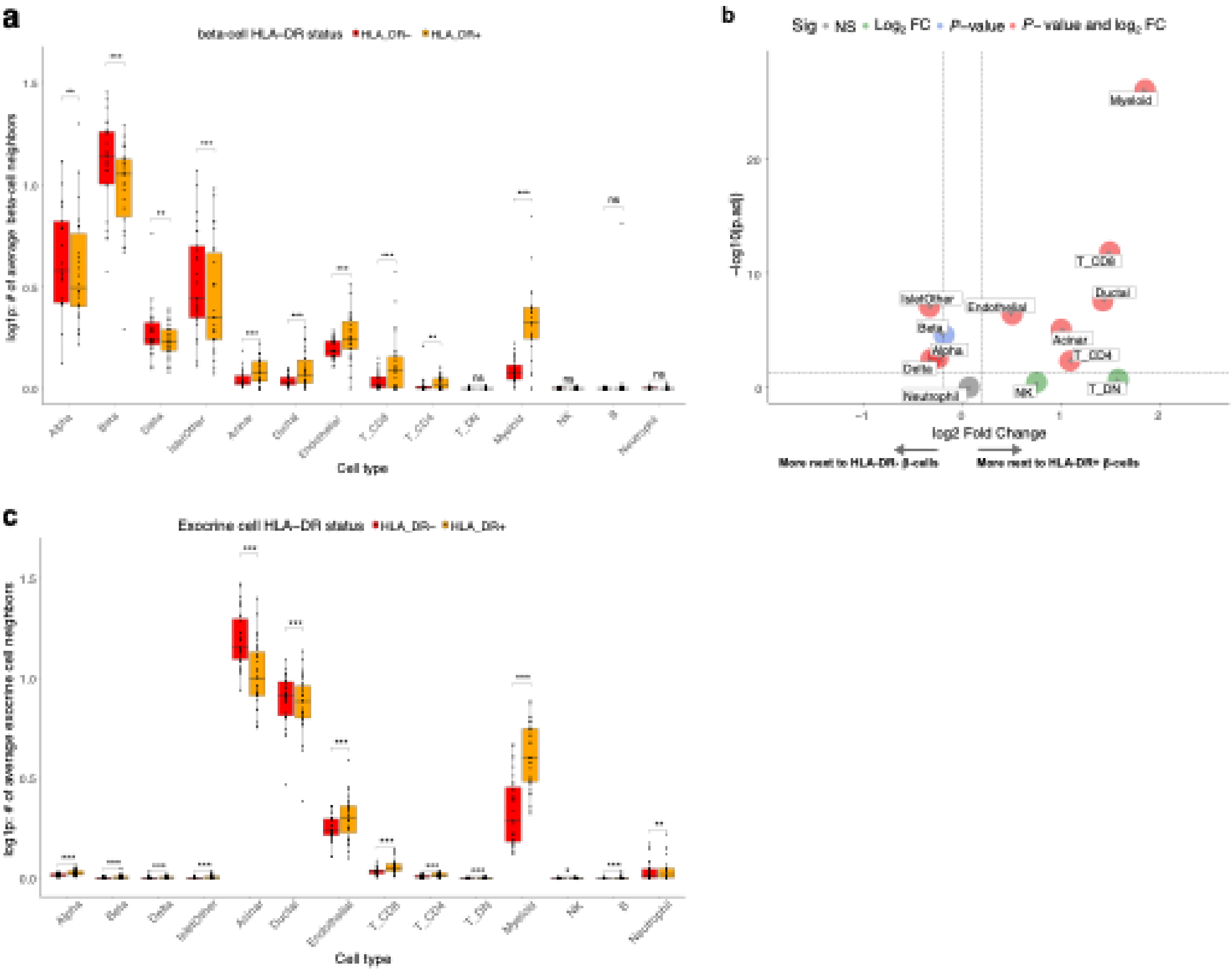

Supplementary Figure 5: *Immune cells are enriched near HLA-DR⁺ cells*. **a:** Average number of neighbors of HLA-DR⁺ and HLA-DR⁻ β-cells from mAAb+ and Onset donors. Proximity is defined based on a spatial nearest-neighbor graph (number of neighbors = 5, maximum distance = 15 μm). Each dot is the number of neighbors by donor, averaged across ROIs**.** The center line indicates the median, the box bounds represent the IQR from the 25th to the 75th percentile, and the whiskers extend to the minimum and maximum values within 1.5 times the IQR. **b:** Volcano plot of differential number of neighbors between HLA-DR⁺ and HLA-DR⁻ β-cells. **c:** Average number of neighbors of HLA-DR⁺ and HLA-DR⁻ exocrine cells as described in panel **(a)**. Tests: * *P* <0.05; ** *P* <0.01; *** *P* <0.001 for all comparisons**.** Linear mixed-effects models were fit to compare HLA-DR⁺ and HLA-DR⁻ cells of the same donor. *P*-values are FDR-adjusted and were computed using two-sided Wald t-test with Satterthwaite approximation. (**a-c**) N = 31, i (ROIs) = 2504.

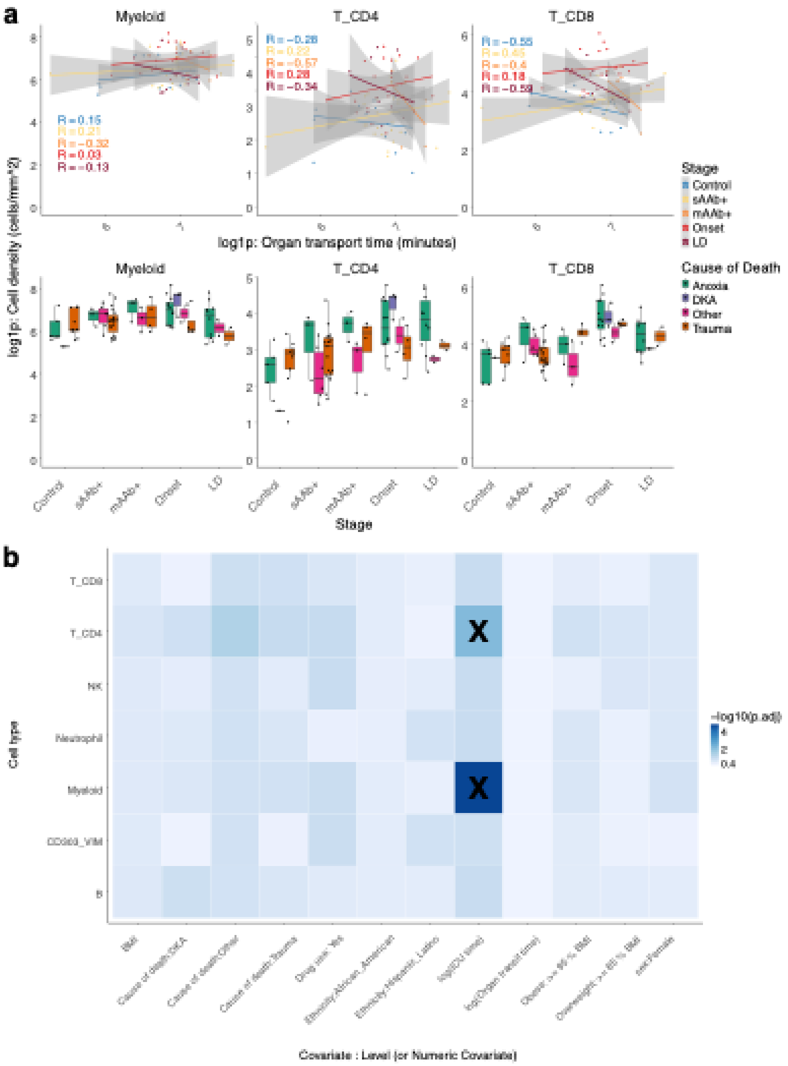

Supplementary Figure 6: *Association of immune cell type densities with covariates.* **a:** The immune cell type densities across disease stages compared against cause of death and organ transit time. Each dot indicates data from one donor with densities averaged across ROIs. The center line indicates the median, the box bounds represent the IQR from the 25th to the 75th percentile, and the whiskers extend to the minimum and maximum values within 1.5 times the IQR. Indicated *R* values are Spearman correlation coefficients. Error bands are the 95 % CIs. **b:** Heatmap of the associations of covariates with densities of all immune cell types. Categorical variables are separated by colons from their respective levels (e.g., 'Drug use:Yes'). Tests: The following linear mixed-effects model were fit: *Cell Density ~ age_group + disease_stage + covariate + (1|case_id)*. Significances were calculated using two-sided Wald t-test with Satterthwaite approximation. FDR-adjusted *P*-values were adjusted separately by covariate to avoid *P*-value deflation. Significant associations are marked with an "X". Control: N=15; sAAb+: N=28; mAAb+: N=10; Onset: 21; LD: N = 14.

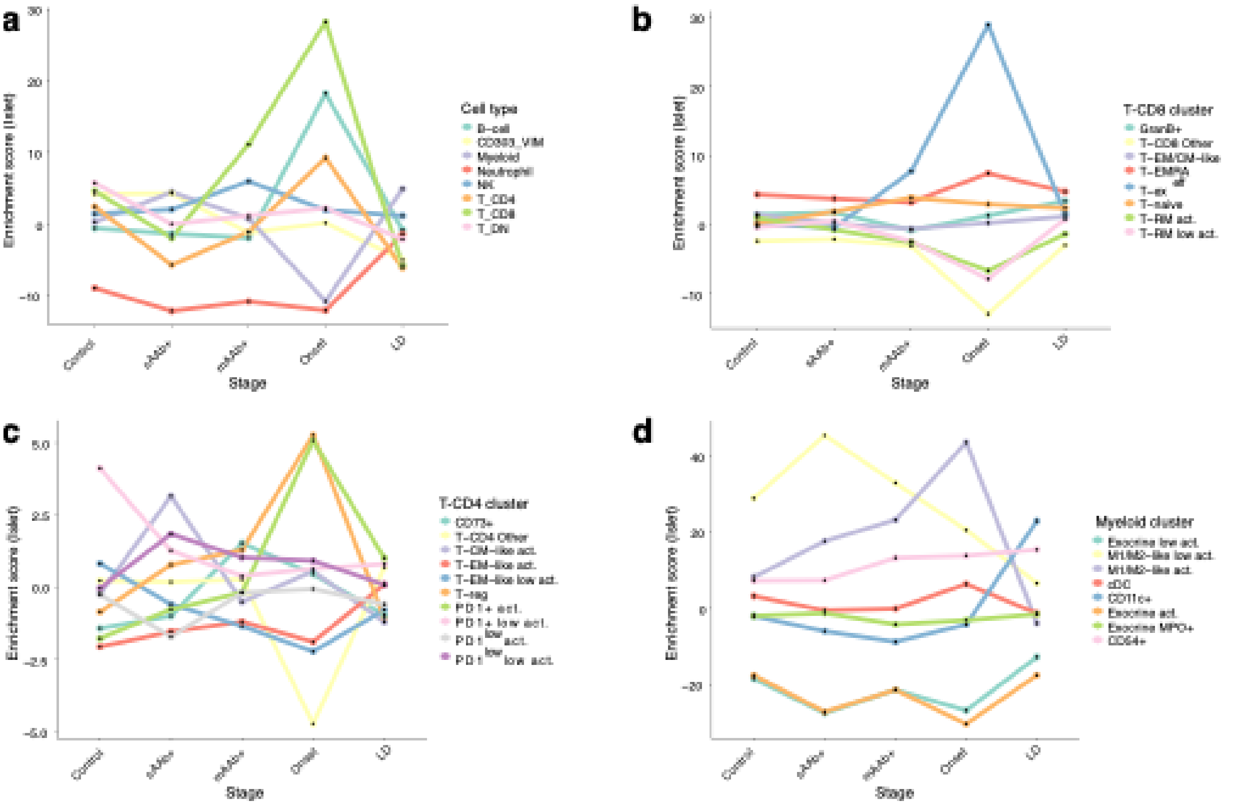

Supplementary Figure 7: *Stage-specific enrichment of immune cells in islets.* Stage-specific enrichment scores of immune cells in distance bins relative to the islet edge, as calculated in Fig. 3f. Only the enrichment scores for islets are shown for **(a)** immune cell types, **(b)** T-CD8 cell subtypes, **(c)** T-CD4 cell subtypes, and **(d)** myeloid subtypes. Enrichment score values are χ^2-residuals.
